# Multi-objective gold rush optimization algorithm: Theoretical Extensions and applications in UAV path planning

**DOI:** 10.1371/journal.pone.0351159

**Published:** 2026-06-10

**Authors:** Kecheng Su, Yaoyang Wang, Yikang Kong, Wenan Liu

**Affiliations:** 1 School of Intelligent Medicine, Chengdu University of Traditional Chinese Medicine, Chengdu, China; 2 School of Mechanical Engineering, University of Science and Technology Beijing (USTB), Beijing, China; 3 School of Air Traffic Management, Civil Aviation University of China, Tianjin, China; 4 Huaian New Bright Imp. & Exp. Trading Co., Ltd., Huai’an City, Jiangsu Province, China; National Taiwan University of Science and Technology, TAIWAN

## Abstract

Multi-objective optimization problems have extensive application value in the fields of engineering and science, among which UAV path planning, as a typical application scenario, has attracted considerable attention. This study innovatively proposes a multi-objective extension of the Gold Rush Optimization algorithm (GRO), namely the Multi-Objective Gold Rush Optimization algorithm (MOGRO). By introducing a reference-point-guided two-level selection mechanism and an external archive strategy, the algorithm effectively addresses the challenge of obtaining Pareto-optimal solutions in multi-objective optimization problems. A systematic validation method is adopted: firstly, a comparative analysis is conducted between MOGRO and seven advanced multi-objective optimization algorithms on a benchmark test set consisting of 18 standard test problems. Subsequently, a UAV path planning multi-objective optimization model is constructed, taking into account both path length and obstacle threats, and the top four algorithms from the benchmark tests are selected for application validation. Experimental results show that the MOGRO algorithm significantly outperforms the comparison algorithms in terms of convergence, distribution, and solution quality, demonstrating excellent optimization performance. This study not only enriches the theoretical system of the GRO algorithm but also provides an innovative solution for UAV path planning in complex environments, with important theoretical value and practical significance.

## 1 Introduction

In recent decades, metaheuristic optimization has attracted considerable attention from researchers in the study and solution of optimization problems. These techniques have been mathematically introduced and made accessible to all, which has accelerated progress in this field [1–3]. These techniques aim to optimize solutions. Overall, metaheuristic optimization strategies can be divided into single-objective and multi-objective optimization. Single-objective techniques such as Genetic Algorithm (GA) [4], Gravitational Search Algorithm (GSA) [5], Differential Evolution (DE) [6], Bat Algorithm (BA) [7], Particle Swarm Optimization [8], Ant Colony Optimization (ACO) [9], Grey Wolf Optimizer (GWO) [10], Goose Optimization Algorithm (GOOSE) [11], Gold Rush Optimization Algorithm (GRO) [12], Sparrow Search Algorithm (SSA) [13], Harris Hawks Optimization (HHO) [14], Snake Optimizer (SO) [15], Gazelle Optimization Algorithm (GOA) [16], Marine Predators Algorithm (MPA) [17], Dung Beetle Optimizer (DBO) [18], Bounty Hunter Optimizer (BHO) [19], Quantum Avian Navigation Algorithm (QANA) [20], and Red-Beaked Blue Magpie Optimizer (RBMO) [21] are some of the recent metaheuristic optimization algorithms that have been widely applied in different fields [22–24]. These methods are designed to provide better and more comprehensive optimization approaches for single-objective problems, but they fail to achieve all objectives. Multi-objective optimization methods aim to deal with multiple conflicting objectives simultaneously in order to find a more optimal solution. They can effectively coordinate the relationships between different objectives and are thus applicable to solving optimization problems in various application scenarios. However, contradictions and inconsistencies often arise in these optimization processes. Three commonly used methods to solve these problems are the a priori method, the a posteriori method, and the interactive method [21,25,26].

The a priori method addresses multi-objective problems by using weight combinations predetermined by the decision-maker. This approach has a simple structure and is easy to implement, but its drawback lies in its difficulty in effectively finding optimal solutions in non-convex objective spaces, and it often requires multiple weight adjustments to gradually approach the ideal solution [27,28]. In contrast, the a posteriori method handles multi-objective optimization problems without aggregating objectives, allowing the generation of multiple solutions in a single run and enabling the decision-maker to select the optimal one from them [29–31]. Ultimately, these solutions form a Pareto front. Although this method involves higher computational costs, it provides more comprehensive and accurate optimization results, making it the most commonly used multi-objective optimization technique. The interactive method, on the other hand, continuously collects and integrates the decision-maker’s preferences during the optimization process to obtain a Pareto-optimal solution that meets the requirements. Despite being less efficient than the other two methods and having difficulty clearly expressing preferences during the optimization process, its advantage lies in maintaining ongoing interaction with the decision-maker, dynamically adjusting the optimization process, and ultimately finding a solution that better meets real-world needs [32,33].

Multi-objective optimization methods take into account individual solutions and the differences that exist between various objectives [34,35]. In order to obtain Pareto-optimal solutions, the preliminary solutions of any multi-objective optimization problem must be distinct. This is because Pareto-optimal solutions need to reflect different characteristics in order to successfully derive the Pareto front, which requires diversity among the solutions. However, there is no guarantee that these solutions will be evenly distributed along the Pareto front, and this may lead to the appearance of complex hypersurfaces on the preliminary Pareto front, especially when there is coordination among multiple objectives [36,37]. In fact, the Pareto front should reflect the relationships among different points on it to enable effective derivation in multi-objective optimization. However, successfully deriving such a solution set presents a significant challenge: the solution set of a multi-objective optimization problem often cannot be expressed in closed form, and in practical applications, the solving process is frequently computationally intensive or even infeasible to implement effectively [38].

In recent decades, multi-objective algorithms have developed rapidly, leading to the emergence of many outstanding algorithms for solving multi-objective problems, such as the Non-dominated Sorting Genetic Algorithm II (NSGA-II) [39], Multi-Objective Particle Swarm Optimization (MOSPO) [40], Strength Pareto Evolutionary Algorithm Version 2 (SPEA2) [41], Non-dominated Sorting Grey Wolf Optimizer (NSGWO) [42], Multi-Objective Grey Wolf Optimizer (MOGWO) [43], Multi-Objective Ant Lion Optimizer (MOALO) [44], Pareto Archived Evolution Strategy (PAES) [45], Multi-Objective Multi-Verse Optimizer (MOMVO) [46], Multi-Objective Dragonfly Algorithm (MODA) [47], Multi-Objective Moth-Flame Optimizer (MOMFO) [48], Non-dominated Sorting Whale Optimization Algorithm (NSWOA) [49], Multi-Objective Arithmetic Optimization Algorithm (MOAOA) [50], Multi-Objective Equilibrium Optimizer (MOEO) [51] and Multi-Objective Evolutionary Algorithm based on Decomposition (MOEA/D) [52].

In the field of UAV applications, multi-objective optimization technologies are playing an increasingly important role. Particularly in complex and hazardous environments, unmanned aerial vehicles (UAVs), with their superior maneuverability, cost-effectiveness, and advantages in travers ability and safety far beyond human capabilities, have been widely applied in real-world scenarios such as disaster rescue, smart agriculture, regional surveillance, and communication maintenance [53]. For example, in disaster rescue operations following earthquakes or floods, the post-disaster environment is often highly complex, severely hindering direct access by rescue personnel to affected areas. UAVs, owing to their small size, low cost, and multifunctionality, can play a critical role in the early stages of emergency response. The optimized design of their flight paths can significantly improve survival rates and mission execution efficiency, making path planning a core component of UAV mission design. Specifically, path planning refers to determining the optimal route for a UAV from a starting point to a target point under given constraints to achieve predefined optimization goals [54,55]. With the rapid expansion of UAV application domains, related research has become a key focus in the field of multi-objective optimization.

With the rapid expansion of UAV application domains, related research has become an important direction in the field of multi-objective optimization. Although several multi-objective optimization methods have been proposed for UAV path planning, according to the No Free Lunch (NFL) theorem [56], no method or algorithm so far has achieved 100% efficiency in solving multi-objective problems. Despite the increasing number of multi-objective optimization algorithms, several challenges remain unresolved, particularly in balancing convergence and diversity, handling complex Pareto front shapes, and ensuring stability across different problem characteristics. Existing extensions of single-objective algorithms to multi-objective scenarios often suffer from uneven solution distribution and premature convergence. Motivated by these limitations, this study proposes a Multi-Objective Gold Rush Optimization (MOGRO) [1] algorithm. The core motivation lies in leveraging the exploration–exploitation balance inherent in the GRO mechanism and enhancing it with a reference-point-guided environmental selection and an external archive strategy. This design aims to improve both convergence accuracy and distribution uniformity, especially for complex and constrained multi-objective problems such as UAV path planning.

In addition, several multi-objective optimization methods (NSGA-II, MOEA/D, NSSSA, NSDBO, NSGWO, NSWOA, and MSSA) are also processed and compared. This study evaluates the efficiency of the proposed algorithm using three performance metrics across 18 benchmark test problems and further validates its performance in a multi-objective UAV 3D path planning application. The experimental results demonstrate that MOGRO is one of the outstanding multi-objective metaheuristic algorithms. It not only effectively solves complex multi-objective problems but also shows excellent performance in UAV path planning, outperforming the other algorithms involved in the comparison on both levels.

The structure of this paper is organized as follows: Section 2 introduces the single-objective and multi-objective versions of the GRO algorithm. Section 3 presents the experimental results and discussion. Section 4 constructs a multi-objective 3D UAV path planning model and conducts a comparative study of algorithm applications. Finally, Section 5 provides the conclusions and directions for future work.

## 2 Gold rush optimizer and multi-objective gold rush optimizer

### 2.1 Gold rush optimizer (GRO)

#### 2.1.1 Gold prospectors modeling.

The GRO algorithm mimics the main events of a gold rush. In the GRO metaheuristic algorithm, explorers play the same role as the population in GA and particles in PSO. The positions of the gold mine explorers are stored in a matrix called $M_{GP}$, represented by Eq. (1). In this equation, $x_{i,j}$ denotes the position of the ithexplorer in the jth dimension. Dim is the dimension size, and N is the number of gold mine explorers.


$$\begin{array}{c} \begin{array}{c} MGP=\left[\begin{array}{ccccc} x\text{1},\text{1} & \cdots & x\text{1},j & \cdots & x\text{1},Dim\\ \vdots & \ddots & \vdots & \mathrm{\backslash rotatebox}90\$\ddots \$ & \vdots \\ xi,\text{1} & \cdots & xi,j & \cdots & xi,Dim\\ \vdots & \mathrm{\backslash rotatebox}90\$\ddots \$ & \vdots & \ddots & \vdots \\ xN,\text{1} & \cdots & xN,j & \cdots & xN,Dim \end{array}\right] \end{array} \end{array}$$ (1)

An objective function is needed to evaluate the gold mine explorers during the optimization process. The evaluation values of the explorers are stored in an evaluation matrix $M_{F}$ according to Eq. (2). In this equation, $x_{i,j}$ represents the position of the ith explorer in the jth dimension, and f is the evaluation function.


$$\begin{array}{c} \begin{array}{c} MF=\left[\begin{array}{ccccc} f(x\text{1},\text{1} & \cdots & x\text{1},j & \cdots & x\text{1},Dim)\\ \vdots & \ddots & \vdots & \mathrm{\backslash rotatebox}90\$\ddots \$ & \vdots \\ f(xi,\text{1} & \cdots & xi,j & \cdots & xi,Dim)\\ \vdots & \mathrm{\backslash rotatebox}90\$\ddots \$ & \vdots & \ddots & \vdots \\ f(xN,\text{1} & \cdots & xN,j & \cdots & xN,Dim) \end{array}\right] \end{array} \end{array}$$ (2)

#### 2.1.2 Migration of prospectors.

After discovering a gold mine, the gold mine explorers migrate to that mine to extract gold. During the execution of the metaheuristic algorithm, the location of the richest gold mine represents the optimal point in the search space. Since its exact position is unknown, the position of the best gold mine explorer is used as an estimate of the optimal gold mine location. The migration of gold mine explorers to the gold mine is modeled using Eqs. (3) and (4).


$$\begin{array}{c} D_{\text{1}}=C_{\text{1}}\times X^{*}(t)-X_{i}(t) \end{array}$$ (3)

$$\begin{array}{c} \begin{array}{c} {Xnew}_{i}(t+\text{1})=X_{i}(t)+A_{\text{1}}\times D_{\text{1}} \end{array} \end{array}$$ (4)

where $X^{*}(t)$, $X_{i}(t)$ and t represent the position of the best gold mine, the position of the ith gold mine explorer, and the current iteration tt, respectively. ${Xnew}_{i}$ denotes the new position of the ith gold mine explorer, while $A_{\text{1}}$ and $C_{\text{1}}$ are vector coefficients calculated by Eqs. (5) and (6).


$$\begin{array}{c} \begin{array}{c} A_{\text{1}}=\text{1}+l_{\text{1}}\left(r_{\text{1}}-\frac{\text{1}}{\text{2}}\right) \end{array} \end{array}$$ (5)

$$\begin{array}{c} \begin{array}{c} C_{\text{1}}=\text{2}\times r_{\text{2}} \end{array} \end{array}$$ (6)

here, $r_{\text{1}}$ and $r_{\text{2}}$ are random vectors with values in the range [0, 1]. $l_{\text{1}}$ is the convergence component defined by Eq. (7); if e equals 1, it decreases linearly from 2 to $\frac{\text{1}}{T}$; for values greater than 1, it decreases nonlinearly. T represents the maximum number of iterations.


$$\begin{array}{c} \begin{array}{c} l_{e}={\left(\frac{T-t}{T-\text{1}}\right)}^{e}\times \left(\text{2}-\frac{\text{1}}{T}\right)+\frac{\text{1}}{T} \end{array} \end{array}$$ (7)

#### 2.1.3 Gold mining.

Each gold mine explorer mines the gold mine area to search for more gold. In mathematical modeling, the position of each gold mine explorer is considered an approximate location of a gold mine. The mathematical relationships related to gold mining are modeled using Eqs. (8) and (9).


$$\begin{array}{c} \begin{array}{c} D_{\text{2}}=X_{i}(t)-X_{r}(t) \end{array} \end{array}$$ (8)

$$\begin{array}{c} \begin{array}{c} {Xnew}_{i}(t+\text{1})=X_{r}(t)+A_{\text{2}}\times D_{\text{2}} \end{array} \end{array}$$ (9)

where $X_{r}$, $X_{i}$, t and ${Xnew}_{i}$ represent the position of a randomly selected gold mine explorer r the position of the gold mine explorer i, the current iterationt, and the new position of the gold mine explorer ii, respectively. $A_{\text{2}}$ is a vector coefficient calculated by Eq. (10).


$$\begin{array}{c} \begin{array}{c} A_{\text{1}}=\text{2}\times l_{\text{2}}\times r_{\text{1}}-l_{\text{2}} \end{array} \end{array}$$ (10)

#### 2.1.4 Collaboration between prospectors.

Since gold mine exploration is sometimes carried out through teamwork, mathematical modeling using Eqs. (11) and (12) is employed to describe the cooperation among gold mine explorers. In this context, $g_{\text{1}}$ and $g_{\text{2}}$ are two randomly selected gold mine explorers. A three-way cooperation is established among gold mine explorers i, $g_{\text{1}}$ and $g_{\text{2}}$; where $D_{\text{3}}$ represents the cooperation vector.


$$\begin{array}{c} \begin{array}{c} D_{\text{3}}=X_{g_{\text{2}}}(t)-X_{g_{\text{1}}}(t) \end{array} \end{array}$$ (11)

$$\begin{array}{c} \begin{array}{c} {Xnew}_{i}(t+\text{1})=X_{i}(t)+r_{\text{1}}\times D_{\text{3}} \end{array} \end{array}$$ (12)

#### 2.1.5 Prospectors relocation.

Gold mine explorers continuously move, and a key parameter in their decision-making process is the pursuit of acquiring more gold. To decide whether an explorer should remain at its current position or move to a new one, these two options are compared using the evaluation function. In this process, if the objective function value improves, the gold mine explorer updates its position; otherwise, it stays in its original position. This is modeled for minimization problems by Eq. (13).


$$\begin{array}{c} \begin{array}{c} @lX_{i}(t+\text{1})=\left\{ \begin{array}{c} @l{Xnew}_{i}(t+\text{1})\;iff\left({Xnew}_{i}(t+\text{1})\right)<f(X_{i}(t))\\ X_{i}(t)\;iff\left({Xnew}_{i}(t+\text{1})\right)\geq f(X_{i}(t)) \end{array}\right. \end{array} \end{array}$$ (13)

### 2.2 Multi-objective gold rush optimizer

#### 2.2.1 Environmental selection.

The environmental selection mechanism in the GRO algorithm constructs a two-level screening framework combining “elite retention + intelligent allocation.” By simulating the competition and cooperation processes in a gold rush, this mechanism integrates non-dominated sorting with reference-point-guided techniques, preserving the convergence advantages of traditional optimization algorithms while effectively addressing the uneven distribution of the Pareto front through a target space partitioning strategy. This design enables the algorithm to adaptively balance convergence and diversity requirements, providing a more comprehensive solution for multi-objective optimization problems.

During the leader selection phase, the mechanism first applies non-dominated sorting to filter high-quality solutions from the population as candidate leaders—analogous to potential high-value mining sites discovered during gold prospecting. Simultaneously, an intelligent allocation technique based on reference points is introduced, which ensures the leader solutions uniformly cover the entire objective space by calculating the match between solutions and reference points. As the algorithm iterates, the distribution of reference points dynamically adjusts to accommodate changes in the shape of the Pareto front, a process similar to prospectors continuously adapting their search strategies based on exploration results.

In the population update phase, a hierarchical screening strategy is employed. First, non-dominated sorting and front ranking are performed on the current population combined with newly generated solutions. Then, combined with GRO-specific operators such as migration, mining, and cooperation, the algorithm enhances search diversity while ensuring convergence. Specifically, the migration operator drives solutions toward optimal regions, the mining operator facilitates local fine-grained search, and the cooperation operator enables information exchange. The reference-point technique plays a key role at this stage by intelligently allocating solutions to ensure an even distribution across the objective space.

This mechanism forms a strong synergy with GRO’s gold rush behavioral model. On one hand, GRO’s stochastic migration and cooperation mechanisms provide a rich pool of candidate solutions for environmental selection; on the other hand, the reference-point guidance in environmental selection ensures search comprehensiveness and efficiency. This synergy allows the algorithm to dynamically adapt to different shapes of the Pareto front, avoiding premature convergence while maintaining optimization efficiency, ultimately achieving an ideal balance between convergence speed and solution set diversity.

#### 2.2.2 External archiving mechanism.

In multi-objective optimization algorithms, the External Archiving Mechanism serves as a key strategy whose core function is to dynamically preserve the set of non-dominated solutions discovered during the optimization process through an auxiliary storage structure independent of the main population. This mechanism optimizes performance via a dual design goal: on one hand, ensuring algorithm convergence; on the other, maintaining broad distribution diversity of the solution set, thereby effectively avoiding local optima traps. Its operational logic is based on real-time archiving principles, wherein the newly generated non-dominated solutions in each generation are compared multidimensionally with the existing archive solutions, executing the following update strategies:

(1) Dominance exclusion: When a new solution is dominated by any solution in the archive, it is automatically filtered out to preserve the elitism of the archive;
(2) Dominance replacement: If a new solution dominates any existing solutions in the archive, a replacement mechanism is triggered to remove the inferior solutions and incorporate the new one;
(3) Non-dominated addition: For new solutions that are mutually non-dominant with archived solutions, the archive is directly expanded to enhance diversity.

To address potential loss of optimal solutions due to archive capacity limits, this mechanism innovatively integrates a dynamic niche regulation technique. When the archive reaches saturation, a density evaluation method based on reference point association is employed: by constructing an Euclidean distance matrix between the solution set and the reference points, solutions located in sparse regions (i.e., those farthest from reference points) are preferentially removed, minimizing disruption to the distribution of the solution set. Simultaneously, an adaptive reference point adjustment algorithm is introduced, allowing the distribution of reference points to dynamically track the morphological evolution of the Pareto front, ensuring that the archive maintenance strategy remains dynamically aligned with front characteristics.

Based on the above mechanism, the pseudocode of MOGRO is shown in Algorithm 1.

Algorithm 1. Pseudo-code of MOGRO.

01: ***Input:*** 𝐝𝐢𝐦,𝐥𝐛,𝐮𝐛,𝐏𝐨𝐩_𝐒𝐢𝐳𝐞(N)Archive_Size,Max_iter,numObj

02: ***Output:*** the best Pareto solution

03: Initialize the population by uniformly sampling candidate solutions

04: Generate a set of reference points Z using the Das-Dennis method

05: Initialize the external archive

06: Calculate the fitness of each Gold miner

07: Selecting high-quality solutions by using Environmental Selection

08: Iter=1

09: **while** Iter≤Max_iter  **do**

10:   Update the best Gold miner $X^{*}(t)$

11:   ***for*** i=1:N **do**

12:  ***Migration of prospectors***

13:   Update the location of the gold prospector after the migration by Eq. (9)

14:  ***Collaboration between prospectors***

15:   Calculate the cooperation vector of the gold mine explorers using Eq. (11)

16:   Update the location of the gold mine explorer after the cooperation by Eq. (12)

17:  ***Prospectors relocation***

18:   Calculate the locations of all gold mine explorers to update the population using Eq. (13)

18:  ***end for***

19:  Merge all populations and evaluate their fitness values

20:  Update high-quality solutions by using Environmental Selection

22:  Update external archive by using External Archiving Mechanism

23: ***end while***

24: Return and save the best Pareto solution

### 2.3 Computational complexity analysis

The computational complexity of the proposed MOGRO algorithm is primarily influenced by the population size N, the number of objectives M, and the maximum number of iterationsT. During each iteration, the algorithm performs several key operations, including solution update, environmental selection, and archive maintenance. Among these, the non-dominated sorting procedure plays a central role in evaluating solution quality, and its worst-case computational cost is $O(MN^{\text{2}})$. In addition, the reference-point-based environmental selection mechanism requires assigning solutions to reference points and performing sorting operations, which introduces an additional computational cost on the order of O(NlogN).

Furthermore, the external archive updating mechanism involves dominance comparisons between newly generated solutions and existing archive members, which may incur a complexity of $O(N^{\text{2}})$ in the worst case. Considering all components, the overall computational complexity of MOGRO can be approximated as $O(T\cdot (MN^{\text{2}}+N^{\text{2}}))$. Although this complexity is comparable to that of classical multi-objective evolutionary algorithms such as NSGA-II, the additional computational overhead introduced by the reference-point guidance and archive mechanism significantly enhances the convergence accuracy and diversity of the obtained Pareto solutions, thereby improving the overall optimization performance of the algorithm.

## 3 Experimental results and analysis

### 3.1 Benchmark problems

This study uses 18 standard multi-objective benchmark problems, including 9 WFG problems and 9 CF problems. The WFG test suite (Walking Fish Group) [57] is designed to evaluate algorithm performance on scalable multi-objective problems with different Pareto front shapes (convex, concave, linear, degenerate) and variable separability. The CF test suite (Constrained Test) [58] focuses on constrained multi-objective problems with complex feasible regions and various constraints. The WFG (Walking Fish Group) and CF (Constrained Functions) test suites are widely used benchmark sets in multi-objective optimization. The WFG suite is designed to evaluate scalability, modality, and separability, while the CF suite focuses on constrained optimization problems. The WFG and CF benchmark suites were selected not only because they are widely used, but also because their problem characteristics are closely aligned with the intended evaluation objectives of MOGRO. Specifically, MOGRO introduces a reference-point-guided environmental selection strategy and an external archive mechanism, which are designed to simultaneously improve convergence, diversity maintenance, and Pareto front coverage. Therefore, benchmark problems with different Pareto front shapes, variable linkages, modality, degeneracy, discontinuity, and constraints are necessary to examine whether these mechanisms are effective under heterogeneous optimization conditions. The WFG suite provides scalable unconstrained problems with convex, concave, discontinuous, degenerate, deceptive, and non-separable properties, which are suitable for testing the ability of MOGRO to maintain a well-distributed solution set under different Pareto front geometries. In contrast, the CF suite introduces constrained feasible regions, nonlinear constraints, mixed Pareto fronts, and deceptive landscapes, which are useful for evaluating the robustness of MOGRO when feasible solutions are difficult to locate and preserve. Thus, the combined use of WFG and CF functions provides a targeted and comprehensive testbed for assessing the main strengths expected from MOGRO, including convergence stability, diversity preservation, archive-based elitism, and adaptability to complex constrained multi-objective problems. The detailed characteristics, including variable correlation, modality, optimization dimension, Pareto front shape, and constraints, are summarized in Table 1. Table 1 briefly outlines various relevant properties of the chosen benchmark problems, including variable correlation, objective characteristics, optimization dimensionality, Pareto front shapes, and the presence or absence of constraints. Since the benchmark problems exhibit different properties, different approaches may be required to solve them. Further information on the characteristics of the benchmark problems used can be found in references [57–59].

**Table 1:** Characteristics of test problems.

Name	Variable Correlation (Separability)	Objective Property (Modality)	Optimization Dimension	Pareto Front Shape	Constraints
**WFG1**	*Separable*	*Unimodal*	*Decision Variables & Objectives*	*Convex, Mixed*	*None*
**WFG2**	*Non-separable*	*f1 Unimodal* *f2 Multimodal*	*Decision Variables & Objectives*	*Convex Discontinuous*	*None*
**WFG3**	*Non-separable*	*Unimodal*	*Decision Variables & Objectives*	*Linear Degenerate*	*None*
**WFG4**	*Non-separable*	*Multimodal*	*Decision Variables & Objectives*	*Concave*	*None*
**WFG5**	*Separable*	*Deceptive*	*Decision Variables & Objectives*	*Concave*	*None*
**WFG6**	*Non-separable*	*Unimodal*	*Decision Variables & Objectives*	*Concave*	*None*
**WFG7**	*Separable*	*Unimodal*	*Decision Variables & Objectives*	*Concave*	*None*
**WFG8**	*Non-separable*	*Unimodal*	*Decision Variables & Objectives*	*Concave*	*None*
**WFG9**	*Non-separable*	*Multimodal Deceptive*	*Decision Variables & Objectives*	*Concave*	*None*
**CF1**	*Partially separable*	*Unimodal*	*Constrained*	*Convex*	*1 Linear constraint*
**CF2**	*Non-separable*	*Multimodal*	*Constrained*	*Concave*	*1 Nonlinear constraint*
**CF3**	*Non-separable*	*Multimodal*	*Constrained*	*Mixed*	*2 Constraints*
**CF4**	*Partially separable*	*Deceptive*	*Constrained*	*Degenerate*	*2 Constraints*
**CF5**	*Non-separable*	*Unimodal*	*Constrained*	*Linear*	*3 Constraints*
**CF6**	*Non-separable*	*Multimodal*	*Constrained*	*Mixed*	*1 Constraint*
**CF7**	*Non-separable*	*Multimodal*	*Constrained*	*Concave*	*2 Constraints*
**CF8**	*Non-separable*	*Multimodal*	*Constrained*	*Mixed*	*3 Constraints*
**CF9**	*Non-separable*	*Deceptive*	*Constrained*	*Degenerate*	*3 Constraints*

Each benchmark problem presents unique challenges. For example, WFG involve multimodal landscapes that test global search ability, while CF problems introduce constraints that evaluate feasibility handling. The performance of MOGRO across these problems demonstrates its robustness in handling diverse optimization difficulties.

### 3.2 The performance metrics

In this section, we compare the performance of MOGRO with other algorithms using three Pareto front-based test metrics: Inverted Generational Distance (IGD) [60], Generational Distance (GD) [61], and Hypervolume Indicator (HV) [62]. Three widely recognized metrics are adopted: IGD, GD, and HV. They are selected for their complementary roles:

- IGD measures both convergence and diversity by calculating distances from the true Pareto front to algorithm solutions.
- GD focuses on convergence accuracy, measuring distances from algorithm solutions to the true front.
- HV evaluates the quality of solution sets by calculating the dominated hypervolume, reflecting both convergence and coverage.

Together, they provide a comprehensive and fair evaluation.

#### 3.2.1 IGD evaluation indicators.

IGD uses the true Pareto front as the reference set and calculates the distance from each reference point on the true Pareto front to the nearest point in the non-dominated solution set obtained by the algorithm. It is often used to measure the convergence of the Pareto front obtained by an algorithm, and its mathematical expression is shown in Eq. (14):


$$\begin{array}{c} \begin{array}{c} IGD=\frac{\text{1}}{n}\sqrt{\sum_{i=\text{1}}^{n}\overset{\text{2}}{\underset{i}{d}}} \end{array} \end{array}$$ (14)

where, n represents the number of true Pareto optimal solutions, and $d_{i}$denotes the Euclidean distance between the i-th solution obtained by the algorithm and the closest member in the true Pareto front. A smaller IGD value indicates that the solution set is closer to the true Pareto front and more evenly distributed.

#### 3.2.2 GD evaluation index.

GD differs from IGD in that IGD takes into account the distribution characteristics of the reference front, while GD only evaluates the distance from the algorithm’s solutions to the reference front. GD is commonly used to assess the quality of the solution set obtained by evolutionary algorithms in multi-objective optimization. GD quantifies the convergence and distribution of the solution set by calculating the degree of approximation between the Pareto front obtained by the algorithm and the pre-set optimal Pareto front. Its mathematical expression is shown in Eq. (15):


$$\begin{array}{c} \begin{array}{c} GD=\sqrt{\frac{\text{1}}{n}\sum_{i=\text{1}}^{n}\overset{\text{2}}{\underset{i}{d}}} \end{array} \end{array}$$ (15)

where, n represents the number of true Pareto optimal solutions, and $d_{i}$ denotes the Euclidean distance between the i-th solution obtained by the algorithm and the closest member in the true Pareto front. A smaller GD value indicates that the algorithm’s solution set is closer to the true Pareto front.

#### 3.2.3 HV evaluation index.

HV, as one of the most widely used performance evaluation metrics in multi-objective optimization, evaluates the quality of the solution set by calculating the volume of the hypercube formed by the non-dominated solution set A obtained by the algorithm in the objective space and the reference point W. The mathematical principle is as follows: For each solution i in the solution set A, a hypercube $V_{i}$ is constructed with the reference point W as a vertex, where solution i serves as the diagonal vertex of the hypercube, and reference point W is the reference vector formed by extracting the worst values from each objective function. The mathematical expression for HV is shown in Eq. (16):


$$\begin{array}{c} \begin{array}{c} HV=volume\left(\overset{\left|\text{A}\right|}{\underset{i=\text{1}}{⋃}}V_{i}\right) \end{array} \end{array}$$ (16)

where, |A| represents the number of non-dominated solutions, and $V_{i}$ denotes the hypercube formed by the i-th non-dominated solution and the reference point. The HV metric evaluates both convergence and diversity: it measures how closely the solution set approximates the true Pareto front (convergence) and reflects the uniformity of the solution distribution (diversity). This metric is sensitive to missing regions of the front, and its computational complexity increases with dimensionality. A larger HV value indicates better solution set quality, but the reference point must be chosen carefully to avoid evaluation bias.

### 3.3 Experimental setting

Table 2 lists the key parameter settings of MOGRO and seven comparison algorithms: NSGAII [39], MOEA/D [63], NSSSA [64],NSDBO [65],NSGWO [42],NSWOA [49] and MSSA [66]. The selected comparison algorithms represent different categories of multi-objective evolutionary algorithms, including dominance-based (NSGA-II), decomposition-based (MOEA/D), and swarm intelligence-based approaches (NSGWO, NSWOA). This selection ensures a fair and comprehensive comparison with state-of-the-art methods.

**Table 2:** Parameter setting.

Algorithm	Parameters
**NSGAII**	*Population size* Np=200*, Archive size* Nr=200*, Max iterations* Iter=100*, Crossover probability* $P_{c}=\text{0}.\text{9}$*, Mutation probability* $P_{m}=\text{0}.\text{1}$*, Crossover distribution index* mu=20*, Polynomial mutation distribution index* mum=20*, Tournament selection size* tour=2
**MOEA/D**	*Population size* Np=200*, Archive size* Nr=200*, Max iterations* Iter=100*, Inertia weight* W=0.4*, Individual learning factor* $C_{\text{1}}=\text{2}$*, Population size* Np=200*, Archive size* Nr=200*, Max iterations* Iter=100*, Number of grid divisions* ngrid=20
**NSSSA**	*Population size* Np=200*, Archive size* Nr=200*, Max iterations* Iter=100*, Producer proportion* $P_{percent}=\text{0}.\text{2}$
**NSDBO**	*Population size* Np=200*, Archive size* Nr=200*, Max iterations* Iter=100*, Convergence factor* R=1−Iteration/Max_iteration*, Global exploration* $p_{num\text{1}}=\text{0}.\text{2}$*, Local exploitation* $p_{num\text{2}}=\text{0}.\text{4}$*, Balanced strategy* $p_{num\text{3}}=\text{0}.\text{4}$
**NSGWO**	*Population size* Np=200*, Archive size* Nr=200*, Max iterations* Iter=100*, Grid inflation coefficient* Q=0.1*, Archive member selection pressure* Y=2*, Number of grids per dimension* ngrid=20*, Leader selection pressure parameter:* β=4
**NSWOA**	*Population size* Np=200*, Archive size* Nr=200*, Max iterations* Iter=100*, Linearly decreasing parameter* a=2−Iteration*(2/Max_iteration)*, Spiral coefficient* b=1*, Spiral parameter* t=(a2−1)*rand+1*, Scaling factor* SF=round(1+rand)
**MSSA**	*Population size* Np=200*, Archive size* Nr=200*, Max iterations* Iter=100*, Number of grid divisions* ngrid=20
**MOGRO**	*Population size* Np=200*, Archive size* Nr=200*, Max iterations* Iter=100*,* $Randomvariablesr_{\text{1}}andr_{\text{2}}\;$

To ensure fairness in the experiments, all algorithms were tested in MATLAB R2023b, with each algorithm independently run 30 times on each test problem using different random seeds. A unified stopping criterion was applied for all algorithms, terminating when the maximum number of iterations was reached. The experimental environment configuration for the algorithm tests is as follows: (1) Processor-13th Gen Intel Core i5-13400 2.50GHz; (2) Memory – 16.00GB; (3) Operating System – Microsoft Windows 11 Professional Edition.

### 3.4 Experimental results

All algorithms were tested using random seeds over 30 independent runs, and the mean and standard deviation of three metrics—IGD, GD, and HV—were recorded. Detailed results are presented in Tables 3–5. For ease of comparative analysis, the mean values of all algorithms on each test problem were ranked, with the best mean and best standard deviation highlighted in bold. It should be noted that a high-quality Pareto front should satisfy the following conditions: the HV metric should be as large as possible, while the IGD and GD metrics should be as small as possible.

**Table 3:** Statistical data of the IGD.

Algorithms		NSGAII	MOEA/D	NSSSA	NSDBO	NSGWO	NSWOA	MSSA	MOGRO
	**Mean**	1.1169E + 00	1.1874E + 00	1.2109E + 00	1.2247E + 00	1.2232E + 00	1.2369E + 00	1.2493E + 00	**1.0667E + 00**
**WFG1**	**Std**	5.4086E-02	1.9334E-02	7.6533E-03	9.6438E-03	9.8215E-03	1.1503E-02	1.7861E-01	1.8566E-02
	**Rank**	2	3	4	6	5	7	8	**1**
	**Mean**	1.8762E-01	1.5844E-02	5.9346E-02	7.9067E-02	7.9273E-02	2.9365E-02	1.6301E-01	**7.7167E-03**
**WFG2**	**Std**	8.1533E-02	4.6162E-03	9.4773E-03	1.5559E-02	1.7655E-02	5.3738E-03	5.6161E-02	**8.5961E-04**
	**Rank**	8	2	4	5	6	3	7	**1**
	**Mean**	4.3656E-02	9.6318E-03	2.7688E-02	4.3180E-02	4.3037E-02	1.7844E-02	3.8843E-01	**6.9298E-03**
**WFG3**	**Std**	1.7926E-02	4.7301E-04	2.2530E-03	4.5311E-03	4.2545E-03	2.0495E-03	2.6536E-01	**2.3677E-04**
	**Rank**	7	2	4	6	5	3	8	**1**
	**Mean**	6.9514E-02	6.8001E-02	6.8803E-02	7.2328E-02	7.1309E-02	6.9734E-02	6.3640E-01	**6.6325E-02**
**WFG4**	**Std**	1.7358E-03	1.6199E-03	1.1715E-03	2.5182E-03	2.6548E-03	1.1421E-03	2.2153E-01	4.4751E-03
	**Rank**	4	2	3	7	6	5	8	**1**
	**Mean**	7.0021E-02	6.7534E-02	6.8449E-02	7.1957E-02	7.0453E-02	6.9961E-02	5.6888E-01	**6.3612E-02**
**WFG5**	**Std**	1.5876E-03	1.8202E-03	1.2171E-03	2.0812E-03	1.5749E-03	1.0379E-03	2.2872E-01	6.6395E-03
	**Rank**	5	2	3	7	6	4	8	**1**
	**Mean**	5.7139E-02	1.1774E-02	3.1811E-02	5.3920E-02	4.7588E-02	1.7775E-02	4.4021E-01	**7.8373E-03**
**WFG6**	**Std**	2.8690E-02	1.2731E-03	4.4666E-03	5.6504E-03	5.1832E-03	4.2812E-03	2.7870E-01	**3.1173E-04**
	**Rank**	7	2	4	6	5	3	8	**1**
	**Mean**	2.1627E-02	1.1207E-02	3.1320E-02	4.3447E-02	4.0025E-02	1.4083E-02	9.1450E-01	**7.6240E-03**
**WFG7**	**Std**	9.0340E-03	1.1055E-03	3.2467E-03	4.4453E-03	3.1019E-03	1.3985E-03	4.7632E-01	**2.4226E-04**
	**Rank**	4	2	5	7	6	3	8	**1**
	**Mean**	2.7589E-01	**2.4380E-01**	2.9655E-01	2.5682E-01	2.5163E-01	3.1848E-01	4.8499E-01	2.6996E-01
**WFG8**	**Std**	4.4492E-02	5.2442E-02	7.9328E-03	2.9980E-02	2.9766E-02	**1.2869E-02**	1.4249E-01	1.3482E-02
	**Rank**	5	**1**	6	3	2	7	8	4
	**Mean**	2.3625E-02	1.8242E-02	2.2120E-02	3.3264E-02	3.1992E-02	2.1527E-02	7.1306E-01	**1.2516E-02**
**WFG9**	**Std**	6.5976E-03	8.8139E-04	1.3289E-03	2.5152E-03	1.9210E-03	1.6746E-03	4.0330E-01	**5.5367E-04**
	**Rank**	5	2	4	7	6	3	8	**1**
	**Mean**	2.7634E-03	2.7940E-03	2.8171E-03	1.1013E-02	4.5918E-03	6.5452E-03	3.4345E-01	**1.9115E-03**
**CF1**	**Std**	3.7474E-04	3.3703E-04	2.6292E-04	3.3524E-03	5.6435E-04	5.3699E-04	1.9032E-01	**1.3225E-04**
	**Rank**	2	3	4	7	5	6	8	**1**
	**Mean**	3.8953E-02	4.2008E-02	5.1289E-02	2.6898E-02	2.3945E-02	3.2951E-02	4.7834E-01	**1.4431E-02**
**CF2**	**Std**	1.7442E-02	6.9621E-03	1.0099E-02	5.8485E-03	4.2348E-03	4.7653E-03	3.4021E-01	**2.9073E-03**
	**Rank**	5	6	7	3	2	4	8	**1**
	**Mean**	5.5896E-01	6.2086E-01	4.7438E-01	3.5193E-01	3.5094E-01	8.0514E-01	1.4121E + 00	**3.3816E-01**
**CF3**	**Std**	1.7316E-01	2.5155E-01	9.2320E-02	**6.4352E-02**	6.6445E-02	2.7639E-01	2.4436E + 00	8.1054E-02
	**Rank**	5	6	4	3	2	7	8	**1**
	**Mean**	8.0218E-02	6.3537E-02	8.0243E-02	4.5217E-02	4.7809E-02	8.7362E-02	1.8154E-01	**4.3119E-02**
**CF4**	**Std**	2.7551E-02	7.2430E-03	1.0529E-02	7.3589E-03	7.7240E-03	1.1139E-02	8.4421E-02	**5.1774E-03**
	**Rank**	7	2	5	3	4	6	8	**1**
	**Mean**	4.5543E-01	3.1288E-01	4.7836E-01	**1.2058E-01**	1.2202E-01	3.4439E-01	3.2828E-01	1.4184E-01
**CF5**	**Std**	1.3206E-01	7.1149E-02	7.7809E-02	**3.3525E-02**	5.2507E-02	8.0063E-02	1.3806E-01	4.4377E-02
	**Rank**	7	4	8	**1**	2	6	5	3
	**Mean**	2.6893E-02	2.7457E-02	4.1669E-02	2.0492E-02	**1.9998E-02**	2.7630E-02	1.3568E-01	2.0650E-02
**CF6**	**Std**	8.2751E-03	7.8018E-03	1.2771E-02	2.1874E-03	2.5135E-03	3.9283E-03	6.7596E-02	**1.7310E-03**
	**Rank**	4	5	7	2	**1**	6	8	3
	**Mean**	2.3079E-01	2.0232E-01	1.9077E-01	1.0416E-01	9.4843E-02	2.8939E-01	4.0575E-01	**7.4565E-02**
**CF7**	**Std**	1.1232E-01	7.4798E-02	7.7335E-02	1.8132E-02	1.8063E-02	9.4082E-02	2.3177E-01	**1.6365E-02**
	**Rank**	6	5	4	3	2	7	8	**1**
	**Mean**	7.3666E-01	1.2955E-01	1.0266E-01	8.9821E-02	8.8126E-02	1.6464E-01	8.4118E-01	**7.4800E-02**
**CF8**	**Std**	4.7960E-01	1.8614E-02	1.0753E-02	8.4780E-03	9.3162E-03	2.0796E-02	6.0145E-01	**7.4791E-03**
	**Rank**	7	5	4	3	2	6	8	**1**
	**Mean**	2.5765E-01	9.3427E-02	7.3919E-02	6.2412E-02	6.1762E-02	9.5204E-02	1.9558E + 00	**5.3714E-02**
**CF9**	**Std**	9.7961E-02	1.5139E-02	5.7958E-03	3.9962E-03	**3.0501E-03**	1.0968E-02	4.2591E + 00	6.5407E-03
	**Rank**	7	5	4	3	2	6	8	**1**

**Table 4:** Statistical data of the GD.

Algorithms		NSGAII	MOEA/D	NSSSA	NSDBO	NSGWO	NSWOA	MSSA	MOGRO
	**Mean**	**7.9258E-02**	1.1795E-01	1.2226E-01	8.7274E-02	8.6907E-02	1.5143E-01	9.1256E-02	8.4330E-02
**WFG1**	**Std**	3.8338E-03	8.1225E-03	8.1536E-03	**4.3219E-04**	5.4809E-04	1.0111E-02	9.5975E-03	6.2810E-03
	**Rank**	**1**	6	7	4	3	8	5	2
	**Mean**	4.3760E-03	4.6573E-04	4.4954E-03	3.6798E-03	3.5330E-03	1.7252E-03	4.9326E-03	**3.6206E-04**
**WFG2**	**Std**	1.2853E-03	8.9483E-05	7.2874E-04	6.2658E-04	5.4092E-04	2.9165E-04	2.7944E-03	**6.3409E-05**
	**Rank**	6	2	7	5	4	3	8	**1**
	**Mean**	3.5269E-03	5.1618E-04	2.1760E-03	3.7553E-03	3.7210E-03	1.2671E-03	4.1870E-03	**2.6690E-04**
**WFG3**	**Std**	1.2935E-03	4.9478E-05	2.3606E-04	4.5271E-04	4.4148E-04	1.7541E-04	1.3579E-03	**2.7012E-05**
	**Rank**	5	2	4	7	6	3	8	**1**
	**Mean**	4.6131E-03	4.5242E-03	4.5270E-03	4.5829E-03	4.5494E-03	4.6683E-03	1.6689E-02	**4.4192E-03**
**WFG4**	**Std**	8.2746E-05	**1.0923E-05**	1.1400E-05	4.1995E-05	2.0575E-05	3.1431E-05	1.2066E-02	3.0470E-04
	**Rank**	6	2	3	5	4	7	8	**1**
	**Mean**	4.6166E-03	4.5233E-03	4.5254E-03	4.5694E-03	4.5468E-03	4.6937E-03	1.5919E-02	**4.2105E-03**
**WFG5**	**Std**	1.0011E-04	1.7155E-05	**1.2798E-05**	1.5876E-05	1.9511E-05	1.7275E-05	1.2903E-02	5.1547E-04
	**Rank**	6	2	3	5	4	7	8	**1**
	**Mean**	4.1507E-03	4.3334E-04	1.6975E-03	4.2317E-03	3.7496E-03	9.9498E-04	4.2976E-03	**2.3692E-04**
**WFG6**	**Std**	2.0903E-03	4.4000E-05	2.7403E-04	4.8929E-04	4.6104E-04	2.9027E-04	1.4813E-03	**3.6885E-05**
	**Rank**	6	2	4	7	5	3	8	**1**
	**Mean**	1.4543E-03	4.1463E-04	1.7030E-03	2.9127E-03	2.6735E-03	6.6982E-04	1.2044E-02	**1.9110E-04**
**WFG7**	**Std**	8.0922E-04	4.3118E-05	1.3819E-04	2.5376E-04	3.3355E-04	6.4691E-05	9.0807E-03	**1.6534E-05**
	**Rank**	4	2	5	7	6	3	8	**1**
	**Mean**	1.9058E-02	**7.2110E-03**	2.3906E-02	1.8697E-02	1.8878E-02	3.6871E-02	2.3226E-02	2.2696E-02
**WFG8**	**Std**	3.8358E-03	3.2430E-03	3.2326E-03	1.7990E-03	**1.2598E-03**	3.2366E-03	3.9025E-03	3.2022E-03
	**Rank**	4	**1**	7	2	3	8	6	5
	**Mean**	1.1234E-03	8.7192E-04	1.1327E-03	1.7945E-03	1.7728E-03	1.1238E-03	2.9018E-03	**3.6743E-04**
**WFG9**	**Std**	5.1042E-04	6.3821E-05	6.4255E-05	1.4573E-04	2.0407E-04	1.1654E-04	2.4789E-03	**4.9819E-05**
	**Rank**	3	2	5	7	6	4	8	**1**
	**Mean**	1.4369E-03	1.4353E-03	1.4424E-03	**1.2445E-03**	1.4660E-03	1.5317E-03	1.5972E-03	1.4443E-03
**CF1**	**Std**	2.0430E-05	1.9622E-05	1.5723E-05	1.6532E-04	2.3746E-05	3.3797E-05	1.2843E-03	**3.0956E-06**
	**Rank**	3	2	4	**1**	6	7	8	5
	**Mean**	1.6668E-02	2.3457E-02	1.2517E-02	1.1384E-02	1.2191E-02	2.9808E-02	**8.3208E-03**	9.9900E-03
**CF2**	**Std**	1.3416E-02	2.1235E-02	6.3012E-03	6.0561E-03	**5.1340E-03**	2.0791E-02	1.1142E-02	1.0001E-02
	**Rank**	6	7	5	3	4	8	**1**	2
	**Mean**	4.2137E-01	3.4547E-01	1.2075E + 00	3.7653E-01	3.4812E-01	1.7469E + 00	3.3479E-01	**1.0562E-01**
**CF3**	**Std**	2.7164E-01	1.7421E-01	8.8278E-01	**6.4572E-02**	9.6145E-02	1.1321E + 00	3.8036E-01	9.0477E-02
	**Rank**	6	3	7	5	4	8	2	**1**
	**Mean**	3.8775E-02	3.5684E-02	4.6370E-02	3.5422E-02	4.1790E-02	1.0358E-01	1.7377E-02	**8.5725E-03**
**CF4**	**Std**	4.3063E-02	4.3952E-02	5.4788E-02	1.3777E-02	1.4047E-02	9.3686E-02	2.3440E-02	**1.2859E-02**
	**Rank**	5	6	7	2	3	8	4	**1**
	**Mean**	9.5878E-02	5.9421E-02	6.0915E-02	**1.5839E-02**	2.5344E-02	4.7623E-01	1.7471E-01	1.8756E-02
**CF5**	**Std**	5.7586E-02	3.4739E-02	4.2539E-02	**1.2130E-02**	2.5640E-02	2.4518E-01	1.5747E-01	1.3450E-02
	**Rank**	6	4	5	**1**	3	8	7	2
	**Mean**	2.2553E-02	2.0078E-02	1.1641E-02	6.8936E-03	6.5841E-03	5.4269E-02	2.8219E-02	**5.0553E-03**
**CF6**	**Std**	2.6333E-02	1.5562E-02	1.5072E-02	9.0119E-03	1.0870E-02	3.9055E-02	8.2932E-02	**8.7093E-03**
	**Rank**	6	5	4	3	2	8	7	**1**
	**Mean**	1.2967E-01	2.4013E-01	1.8230E-01	1.2091E-01	1.1634E-01	6.1923E-01	8.4261E-02	**2.9365E-02**
**CF7**	**Std**	1.2550E-01	3.2581E-01	1.5805E-01	3.7330E-02	**3.4693E-02**	5.9708E-01	9.0999E-02	4.7771E-02
	**Rank**	5	7	6	4	3	8	2	**1**
	**Mean**	1.0104E + 00	2.0612E-01	9.8357E-02	1.3320E-01	1.9415E-01	1.1774E + 00	5.9877E-01	**3.3415E-02**
**CF8**	**Std**	4.2604E-01	2.2030E-01	1.0680E-01	9.3355E-02	8.3466E-02	3.6237E-01	6.1739E-01	**4.5485E-02**
	**Rank**	7	5	2	3	4	8	6	**1**
	**Mean**	3.1844E-01	1.2026E-01	4.0636E-02	8.1233E-02	8.2676E-02	2.6932E-01	2.2682E-01	**2.3431E-02**
**CF9**	**Std**	1.9110E-01	8.7291E-02	2.7492E-02	5.5920E-02	4.1225E-02	1.1987E-01	3.6826E-01	**2.6390E-02**
	**Rank**	8	5	2	3	4	7	6	**1**

**Table 5:** Statistical data of the HV.

Algorithms		NSGAII	MOEA/D	NSSSA	NSDBO	NSGWO	NSWOA	MSSA	MOGRO
	**Mean**	2.1476E-01	1.8828E-01	1.8306E-01	1.6568E-01	1.6540E-01	1.6803E-01	1.6311E-01	**2.3349E-01**
**WFG1**	**Std**	2.2055E-02	4.9920E-03	3.7711E-03	4.0665E-03	**3.0927E-03**	3.0686E-03	5.1954E-02	6.1046E-03
	**Rank**	2	3	4	6	7	5	8	**1**
	**Mean**	5.9085E-01	6.3115E-01	6.1510E-01	6.1217E-01	6.1139E-01	6.2521E-01	5.6500E-01	**6.3236E-01**
**WFG2**	**Std**	1.0047E-02	1.0170E-03	2.3228E-03	3.3513E-03	3.5891E-03	1.3752E-03	2.1971E-02	**6.3468E-04**
	**Rank**	7	2	4	5	6	3	8	**1**
	**Mean**	5.6171E-01	5.8148E-01	5.7093E-01	5.5829E-01	5.5947E-01	5.7659E-01	4.5214E-01	**5.8311E-01**
**WFG3**	**Std**	9.4978E-03	3.3150E-04	1.3689E-03	2.9963E-03	2.6792E-03	1.1601E-03	6.3248E-02	**2.0144E-04**
	**Rank**	5	2	4	7	6	3	8	**1**
	**Mean**	3.0902E-01	3.1053E-01	3.0897E-01	3.0778E-01	3.0801E-01	3.0856E-01	1.7641E-01	**3.1078E-01**
**WFG4**	**Std**	8.4542E-04	2.4289E-03	8.6419E-04	1.2091E-03	1.2755E-03	**7.9622E-04**	2.5693E-02	2.5042E-03
	**Rank**	3	2	4	7	6	5	8	**1**
	**Mean**	3.0865E-01	3.1021E-01	3.0934E-01	3.0787E-01	3.0816E-01	3.0842E-01	1.9780E-01	**3.1210E-01**
**WFG5**	**Std**	**3.5955E-04**	1.5985E-03	7.9231E-04	1.0797E-03	9.1083E-04	7.5120E-04	2.9963E-02	3.4528E-03
	**Rank**	4	2	3	7	6	5	8	1
	**Mean**	3.1977E-01	3.4671E-01	3.3794E-01	3.1906E-01	3.2327E-01	3.4322E-01	2.4928E-01	**3.4777E-01**
**WFG6**	**Std**	1.5139E-02	3.3011E-04	1.4009E-03	3.3001E-03	2.9809E-03	1.7198E-03	3.7174E-02	**3.1607E-04**
	**Rank**	6	2	4	7	5	3	8	**1**
	**Mean**	3.3949E-01	3.4700E-01	3.3827E-01	3.2749E-01	3.3017E-01	3.4525E-01	1.7396E-01	**3.4816E-01**
**WFG7**	**Std**	5.4359E-03	2.5702E-04	9.2747E-04	2.8960E-03	1.6257E-03	4.5296E-04	7.1731E-02	**1.7200E-04**
	**Rank**	4	2	5	7	6	3	8	**1**
	**Mean**	2.3523E-01	**2.9026E-01**	2.3207E-01	2.3170E-01	2.3314E-01	2.2007E-01	1.9622E-01	2.4091E-01
**WFG8**	**Std**	9.1649E-03	4.6349E-03	1.6953E-03	8.9107E-03	9.3884E-03	3.8944E-03	1.1943E-02	**1.0907E-03**
	**Rank**	3	**1**	5	6	4	7	8	2
	**Mean**	3.3394E-01	3.3719E-01	3.3519E-01	3.2967E-01	3.3028E-01	3.3538E-01	2.0693E-01	**3.4030E-01**
**WFG9**	**Std**	3.8001E-03	5.6952E-04	5.3312E-04	1.3710E-03	9.7552E-04	8.5433E-04	5.0845E-02	**4.6668E-04**
	**Rank**	5	2	4	7	6	3	8	**1**
	**Mean**	5.8235E-01	5.8211E-01	5.8218E-01	5.6908E-01	5.7958E-01	5.7646E-01	3.1105E-01	**5.8341E-01**
**CF1**	**Std**	1.3937E-04	4.1872E-04	2.0772E-04	5.3033E-03	7.4121E-04	6.7104E-04	1.1479E-01	**9.4970E-05**
	**Rank**	2	4	3	7	5	6	8	**1**
	**Mean**	6.6752E-01	6.6206E-01	6.6166E-01	6.8925E-01	6.9220E-01	6.7822E-01	3.4011E-01	**7.0674E-01**
**CF2**	**Std**	1.6063E-02	8.8198E-03	1.2998E-02	7.1953E-03	4.0468E-03	5.5030E-03	1.5531E-01	**2.5588E-03**
	**Rank**	5	6	7	3	2	4	8	**1**
	**Mean**	3.6384E-02	3.1998E-02	1.4449E-02	4.3297E-02	4.0016E-02	6.2244E-03	1.8304E-02	**8.3939E-02**
**CF3**	**Std**	3.9053E-02	4.2129E-02	2.1875E-02	3.6485E-02	4.0613E-02	**1.2124E-02**	3.4066E-02	4.4964E-02
	**Rank**	4	5	7	2	3	8	6	1
	**Mean**	4.5114E-01	4.6852E-01	4.4981E-01	5.0288E-01	4.9761E-01	4.3177E-01	3.2835E-01	**5.2987E-01**
**CF4**	**Std**	3.1869E-02	1.6879E-02	1.6377E-02	1.0442E-02	1.1806E-02	1.8882E-02	7.8571E-02	**1.0116E-02**
	**Rank**	7	5	4	2	3	6	8	**1**
	**Mean**	9.9478E-02	2.0325E-01	1.4302E-01	**3.9736E-01**	3.9156E-01	1.1323E-01	1.9185E-01	3.8385E-01
**CF5**	**Std**	8.5715E-02	5.5360E-02	**2.1657E-02**	4.2272E-02	6.7904E-02	6.6019E-02	1.0839E-01	4.7532E-02
	**Rank**	8	4	6	**1**	2	7	5	3
	**Mean**	6.9513E-01	6.7864E-01	6.5885E-01	7.0937E-01	7.0853E-01	6.8042E-01	5.7846E-01	**7.1405E-01**
**CF6**	**Std**	9.5093E-03	6.1097E-03	1.1632E-02	1.9845E-03	1.8048E-03	6.8654E-03	4.6100E-02	**1.5880E-03**
	**Rank**	4	6	7	2	3	5	8	**1**
	**Mean**	4.0601E-01	4.2236E-01	4.2355E-01	5.5236E-01	5.6621E-01	2.8909E-01	2.4897E-01	**6.1293E-01**
**CF7**	**Std**	1.3836E-01	1.0181E-01	1.0357E-01	2.9952E-02	2.3876E-02	1.1797E-01	1.4474E-01	**2.1033E-02**
	**Rank**	6	5	4	3	2	7	8	**1**
	**Mean**	8.5237E-02	4.3362E-01	4.6201E-01	4.9163E-01	4.9532E-01	3.6060E-01	6.5238E-02	**4.9839E-01**
**CF8**	**Std**	9.4967E-02	2.8096E-02	2.2402E-02	1.0975E-02	1.0814E-02	3.6509E-02	5.2242E-02	**1.0441E-02**
	**Rank**	7	5	4	3	2	6	8	**1**
	**Mean**	2.5828E-01	4.5325E-01	4.7240E-01	5.0843E-01	5.1265E-01	4.6263E-01	6.8890E-02	**5.2001E-01**
**CF9**	**Std**	**1.0234E-01**	2.3689E-02	1.3759E-02	1.0943E-02	1.0569E-02	1.8201E-02	6.8315E-02	1.1824E-02
	**Rank**	7	6	4	3	2	5	8	**1**

Analysis of the IGD metric in Table 3 shows that MOGRO stands out as the most competitive algorithm, achieving the best rank in 15 out of 18 test problems. MOEA/D, NSDBO, and NSGWO each attained the best rank only once, while the remaining five algorithms did not achieve the best rank in any test case under IGD. Examining the WFG, UF, and CF benchmark functions individually: in the WFG benchmark set, MOGRO did not achieve the best IGD performance only on WFG8, where the top three performers were MOEA/D, NSGWO, and NSDBO, respectively. However, based on the Pareto front distribution plots, MOGRO also exhibits a well-distributed front on WFG8, indicating strong performance despite not having the best numerical IGD value. In the CF benchmark set, MOGRO did not achieve the best IGD performance on CF5 and CF6, but still ranked third among all eight compared algorithms, just behind NSDBO and NSGWO.

Analysis of the GD metric in Table 4 indicates that MOGRO achieved the best rank in 13 out of 18 test problems, once again emerging as the most competitive algorithm. NSDBO obtained the best rank in 2 problems, while NSGAII, MOEA/D, and MSSA each achieved it once. Looking at the WFG and CF benchmark sets: for the WFG benchmarks, MOGRO ranked second on WFG1 (slightly behind NSGAII) and fifth on WFG8, but still performed excellently overall, maintaining strong competitiveness across the full WFG test suite. In the CF benchmarks, MOGRO did not secure the best GD rank on CF1, CF2, and CF5, placing fifth, second, and second respectively. Nevertheless, it achieved the best GD rank on the remaining CF functions, confirming its strong overall performance in the CF set.

Table 5’s HV metric analysis reveals that MOGRO achieved the best rank in 16 out of 18 test problems, again demonstrating its dominance. MOEA/D and NSDBO each achieved the best rank only once, while the other five algorithms did not attain a best rank in any case. Looking into the WFG and CF benchmarks: except for WFG8 and CF5, where MOGRO did not achieve the top HV rank, it secured the best HV performance on all remaining 16 benchmark functions. Although it ranked second on UF8 and third on CF5, these results still highlight MOGRO as a highly competitive and leading algorithm.

From the results, it can be observed that MOGRO consistently achieves superior performance across most benchmark problems. This indicates that the proposed mechanisms effectively balance convergence and diversity. In particular, the reference-point-guided selection improves distribution, while the archive mechanism enhances convergence stability.

Table 6 presents the average ranks (in parentheses) of all algorithms across the four metrics, along with the overall ranking results obtained through the Friedman test [67]. The Friedman test results clearly show that the proposed MOGRO is the most competitive algorithm, achieving the best rank in all three key metrics: IGD, GD, and HV. NSWOA follows closely, securing the second-best rank in all three metrics.

**Table 6:** The average rankings of the algorithms for all the metrics.

Algorithms	Ranking (IGD)	Ranking (GD)	Ranking (HV)
**MOEA/D**	6 (5.333)	8 (6.741)	6 (5.259)
**MSSA**	8 (7.481)	6 (5.778)	8 (7.370)
**NSGAII**	7 (5.926)	7 (5.926)	7 (5.741)
**NSDBO**	4 (4.333)	5 (4.407)	4 (4.259)
**NSGWO**	5 (4.407)	3 (4.000)	5 (4.556)
**NSSSA**	2 (3.704)	4 (4.037)	3 (3.778)
**NSWOA**	2 (3.704)	2 (3.667)	2 (3.741)
**MOGRO**	**1 (1.111)**	**1 (1.444)**	**1 (1.074)**

The overall ranking shows MOGRO > NSWOA > NSSSA > NSGWO > NSDBO > MOPSO > NSGAII > MSSA. MOGRO excels especially on deceptive, multimodal, and constrained benchmarks, indicating strong exploration and constraint-handling ability. In practical terms, this means MOGRO can provide more reliable and high-quality solutions for complex engineering tasks such as UAV path planning.

To comprehensively validate the proposed MOGRO, the Wilcoxon rank-sum test [68] was employed to assess whether the results obtained by MOGRO differ significantly from those of the other algorithms at a 5% significance level. The null hypothesis $H_{\text{0}}$ assumes no significant difference between the algorithms: if the p-value is less than 0.05 (i.e., P < 5%), the null hypothesis is rejected, indicating statistical significance; if the p-value is greater than 0.05 (i.e., P > 5%), the null hypothesis is accepted, suggesting no statistically significant performance difference. Tables 7–9 present the test results for all comparison algorithms across the evaluation metrics, where statistically significant results are marked with “+” and non-significant results are marked with “−”. The summary of significance counts is provided at the end of each table.

**Table 7:** Wilcoxon’s rank sum test results of MOGRO vs other algorithms for IGD indicators.

MOGRO vs	NSGAII		MOEA/D		NSSSA		NSDBO		NSGWO		NSWOA		MSSA	
	p-value	Sig.	p-value	Sig.	p-value	Sig.	p-value	Sig.	p-value	Sig.	p-value	Sig.	p-value	Sig.
**WFG1**	8.29E-06	+	3.02E-11	+	3.02E-11	+	3.02E-11	+	3.02E-11	+	3.02E-11	+	3.02E-11	+
**WFG2**	3.02E-11	+	3.02E-11	+	4.50E-11	+	3.02E-11	+	3.02E-11	+	3.02E-11	+	3.02E-11	+
**WFG3**	3.02E-11	+	3.02E-11	+	3.02E-11	+	3.02E-11	+	3.02E-11	+	3.02E-11	+	3.02E-11	+
**WFG4**	2.23E-09	+	9.83E-08	+	1.91E-02	+	1.19E-06	+	3.02E-11	+	8.48E-09	+	3.02E-11	+
**WFG5**	8.84E-07	+	2.78E-07	+	1.52E-03	+	1.36E-07	+	3.82E-10	+	3.69E-11	+	3.02E-11	+
**WFG6**	3.02E-11	+	3.02E-11	+	3.02E-11	+	3.02E-11	+	3.02E-11	+	3.02E-11	+	3.02E-11	+
**WFG7**	3.02E-11	+	3.02E-11	+	3.02E-11	+	3.02E-11	+	3.02E-11	+	3.02E-11	+	3.02E-11	+
**WFG8**	5.49E-01	–	5.49E-11	+	4.83E-01	–	3.16E-10	+	3.51E-02	+	1.37E-03	+	3.02E-11	+
**WFG9**	7.39E-11	+	3.02E-11	+	3.02E-11	+	3.02E-11	+	3.02E-11	+	3.02E-11	+	3.02E-11	+
**CF1**	3.69E-11	+	3.02E-11	+	3.69E-11	+	3.02E-11	+	3.02E-11	+	3.02E-11	+	3.02E-11	+
**CF2**	3.69E-11	+	3.02E-11	+	3.02E-11	+	3.02E-11	+	8.15E-11	+	2.61E-10	+	3.02E-11	+
**CF3**	8.29E-06	+	9.92E-11	+	6.28E-06	+	2.57E-07	+	4.38E-01	–	6.95E-01	–	1.31E-08	+
**CF4**	7.77E-09	+	3.02E-11	+	3.34E-11	+	3.02E-11	+	2.90E-01	–	2.42E-02	+	3.02E-11	+
**CF5**	3.69E-11	+	4.50E-11	+	1.09E-10	+	3.02E-11	+	7.98E-02	–	5.75E-02	–	2.03E-09	+
**CF6**	3.37E-04	+	6.12E-10	+	4.35E-05	+	3.02E-11	+	7.51E-01	–	1.33E-01	–	3.02E-11	+
**CF7**	4.98E-11	+	3.02E-11	+	6.70E-11	+	3.02E-11	+	9.06E-08	+	6.36E-05	+	3.02E-11	+
**CF8**	3.02E-11	+	3.02E-11	+	1.09E-10	+	3.82E-09	+	4.80E-07	+	2.49E-06	+	3.02E-11	+
**CF9**	3.02E-11	+	3.02E-11	+	4.08E-11	+	4.20E-10	+	8.35E-08	+	1.36E-07	+	3.02E-11	+
**+∕ −**	**17/1**		**18/0**		**17/1**		**18/0**		**14/4**		**15/3**		**18/0**	

**Table 8:** Wilcoxon’s rank sum test results of MOGRO vs other algorithms for GD indicators.

MOGRO vs	NSGAII		MOEA/D		NSSSA		NSDBO		NSGWO		NSWOA		MSSA	
	p-value	Sig.	p-value	Sig.	p-value	Sig.	p-value	Sig.	p-value	Sig.	p-value	Sig.	p-value	Sig.
**WFG1**	1.77E-03	+	3.02E-11	+	3.02E-11	+	3.02E-11	+	1.60E-03	+	4.43E-03	+	1.44E-03	+
**WFG2**	3.02E-11	+	3.02E-11	+	3.81E-07	+	3.02E-11	+	3.02E-11	+	3.02E-11	+	3.02E-11	+
**WFG3**	3.02E-11	+	3.02E-11	+	3.02E-11	+	3.02E-11	+	3.02E-11	+	3.02E-11	+	3.02E-11	+
**WFG4**	3.16E-10	+	3.02E-11	+	8.77E-02	–	3.40E-01	–	3.02E-11	+	1.09E-05	+	3.02E-11	+
**WFG5**	4.50E-11	+	3.02E-11	+	3.02E-11	+	9.12E-01	–	1.96E-10	+	1.61E-06	+	3.02E-11	+
**WFG6**	3.02E-11	+	3.02E-11	+	3.69E-11	+	3.02E-11	+	3.02E-11	+	3.02E-11	+	3.02E-11	+
**WFG7**	3.02E-11	+	3.02E-11	+	3.02E-11	+	3.02E-11	+	3.02E-11	+	3.02E-11	+	3.02E-11	+
**WFG8**	8.66E-05	+	3.02E-11	+	3.02E-11	–	3.03E-02	+	1.86E-06	+	1.39E-06	+	2.28E-01	–
**WFG9**	3.82E-09	+	3.02E-11	+	3.02E-11	+	3.02E-11	+	3.02E-11	+	3.02E-11	+	3.02E-11	+
**CF1**	6.57E-02	–	3.02E-11	+	3.02E-11	+	7.51E-01	–	4.98E-04	+	6.77E-05	+	7.73E-02	–
**CF2**	3.67E-03	+	7.09E-08	+	3.02E-11	+	2.13E-05	+	1.95E-03	+	1.25E-04	+	3.11E-01	–
**CF3**	3.08E-08	+	4.08E-11	+	1.84E-05	+	4.50E-11	+	5.57E-10	+	1.17E-09	+	2.60E-05	+
**CF4**	6.53E-07	+	1.96E-10	+	3.02E-11	+	4.57E-09	+	1.69E-09	+	1.07E-09	+	1.24E-03	+
**CF5**	3.82E-10	+	3.02E-11	+	8.15E-11	+	2.15E-06	+	2.17E-01	–	5.01E-01	–	2.67E-09	+
**CF6**	2.53E-04	+	1.41E-09	+	3.02E-11	+	7.96E-03	+	4.20E-01	–	9.94E-01	–	1.19E-01	–
**CF7**	2.57E-07	+	1.09E-10	+	3.02E-11	+	3.35E-08	+	2.23E-09	+	5.46E-09	+	4.94E-05	+
**CF8**	3.02E-11	+	3.02E-11	+	1.61E-10	+	1.41E-04	+	1.25E-07	+	6.72E-10	+	5.09E-08	+
**CF9**	4.98E-11	+	4.08E-11	+	3.69E-11	+	2.27E-03	+	8.20E-07	+	1.16E-07	+	6.67E-03	+
**+∕ −**	**17/1**		**18/0**		**16/2**		**15/3**		**16/2**		**16/2**		**14/4**	

**Table 9:** Wilcoxon’s rank sum test results of MOGRO vs other algorithms for HV indicators.

MOGRO vs	NSGAII		MOEA/D		NSSSA		NSDBO		NSGWO		NSWOA		MSSA	
	p-value	Sig.	p-value	Sig.	p-value	Sig.	p-value	Sig.	p-value	Sig.	p-value	Sig.	p-value	Sig.
**WFG1**	6.77E-05	+	3.02E-11	+	3.02E-11	+	3.02E-11	+	3.02E-11	+	3.02E-11	+	3.02E-11	+
**WFG2**	3.02E-11	+	3.02E-11	+	3.81E-07	+	3.02E-11	+	3.02E-11	+	3.02E-11	+	3.02E-11	+
**WFG3**	3.02E-11	+	3.02E-11	+	3.02E-11	+	3.02E-11	+	3.02E-11	+	3.02E-11	+	3.02E-11	+
**WFG4**	1.01E-08	+	3.96E-08	+	8.77E-02	–	2.38E-07	+	1.21E-10	+	1.01E-08	+	3.02E-11	+
**WFG5**	3.02E-11	+	3.02E-11	+	3.02E-11	+	3.02E-11	+	3.02E-11	+	3.02E-11	+	3.02E-11	+
**WFG6**	3.02E-11	+	3.02E-11	+	3.69E-11	+	3.02E-11	+	3.02E-11	+	3.02E-11	+	3.02E-11	+
**WFG7**	3.02E-11	+	3.02E-11	+	3.02E-11	+	3.02E-11	+	3.02E-11	+	3.02E-11	+	3.02E-11	+
**WFG8**	1.49E-06	+	3.02E-11	+	3.02E-11	–	3.02E-11	+	1.16E-07	+	7.66E-05	+	3.02E-11	+
**WFG9**	4.20E-10	+	3.02E-11	+	3.02E-11	+	3.02E-11	+	3.02E-11	+	3.02E-11	+	3.02E-11	+
**CF1**	3.02E-11	+	3.02E-11	+	3.02E-11	+	3.02E-11	+	3.02E-11	+	3.02E-11	+	3.02E-11	+
**CF2**	3.02E-11	+	3.02E-11	+	3.02E-11	+	3.02E-11	+	3.02E-11	+	3.02E-11	+	3.02E-11	+
**CF3**	4.00E-05	+	6.30E-11	+	1.84E-05	+	4.14E-09	+	4.22E-04	+	2.37E-04	+	2.76E-08	+
**CF4**	3.69E-11	+	3.02E-11	+	3.02E-11	+	3.02E-11	+	3.47E-10	+	6.70E-11	+	3.02E-11	+
**CF5**	4.48E-11	+	3.02E-11	+	8.15E-11	+	3.02E-11	+	3.18E-01	–	3.33E-01	–	5.07E-10	+
**CF6**	3.02E-11	+	3.02E-11	+	3.02E-11	+	3.02E-11	+	2.15E-10	+	1.33E-10	+	3.02E-11	+
**CF7**	3.02E-11	+	3.02E-11	+	3.02E-11	+	3.02E-11	+	4.62E-10	+	5.00E-09	+	3.01E-11	+
**CF8**	2.72E-11	+	3.02E-11	+	1.61E-10	+	1.86E-09	+	3.27E-02	+	4.38E-01	–	2.92E-11	+
**CF9**	3.02E-11	+	3.34E-11	+	3.69E-11	+	3.69E-11	+	3.01E-04	+	1.22E-02	+	2.72E-11	+
**+∕ −**	**18/0**		**18/0**		**16/2**		**18/0**		**17/1**		**17/1**		**18/0**	

In the IGD metric test (Table 7), MOGRO shows statistically significant differences on all 18 problems when compared with MOEA/D, MSSA, and NSDBO. Against NSGAII, NSSSA, NSGWO, and NSWOA, statistically significant differences were observed in 17, 17, 14, and 15 of the 18 problems, respectively. For the GD metric (Table 8), MOGRO differed significantly from the seven algorithms in 17, 18, 16, 15, 16, 16, and 14 problems, respectively. In terms of the HV metric (Table 9), MOGRO demonstrated statistically significant differences in 18, 18, 16, 18, 17, 17, and 18 problems, respectively, when compared to the same set of algorithms. These results strongly indicate that MOGRO performs significantly differently from the seven mainstream algorithms.

A more problem-specific interpretation can further explain the observed performance differences. For WFG2, WFG3, WFG6, WFG7, and WFG9, MOGRO obtains highly competitive or best results in IGD, GD, and HV. These functions involve discontinuity, degeneracy, non-separability, or deceptive multimodal landscapes. The good performance of MOGRO on these problems indicates that the stochastic migration and cooperation operators can effectively enhance exploration, while the external archive preserves high-quality non-dominated solutions once they are found. In addition, the reference-point-guided selection mechanism helps distribute candidate solutions across different regions of the Pareto front, which explains the strong HV values obtained on most WFG functions.

However, the performance on WFG8 is relatively weaker than on other WFG problems. WFG8 is a non-separable problem with strong parameter dependency, where the decision variables are highly correlated. In this case, the movement of one variable may influence the effectiveness of other variables, making it more difficult for population-based search operators to independently refine solutions. Although MOGRO still maintains a competitive Pareto front distribution, its IGD and GD ranks are lower than those of MOEA/D and some other algorithms. This suggests that decomposition-based search may have an advantage on WFG8 because it can decompose the objective space into subproblems and perform more stable local refinement under strong variable dependency. Therefore, WFG8 reveals a potential limitation of MOGRO in handling highly coupled decision-variable structures.

For the CF benchmark suite, MOGRO performs particularly well on CF2, CF3, CF4, CF7, CF8, and CF9, which contain nonlinear constraints, mixed Pareto front shapes, multimodality, and deceptive properties. These results indicate that the archive mechanism helps retain feasible and high-quality non-dominated solutions, while reference-point guidance prevents the population from collapsing into a narrow feasible region. Nevertheless, on CF5, MOGRO does not achieve the best performance. CF5 is characterized by non-separability, a linear Pareto front, and multiple constraints. The relatively weaker performance on this problem may be caused by the interaction between constraint boundaries and the archive update process. When feasible regions are narrow and linearly structured, algorithms such as NSDBO and NSGWO may more directly exploit feasible boundary regions, whereas MOGRO tends to preserve broader diversity. This diversity-oriented behavior is beneficial in most complex problems, but it may slightly reduce convergence efficiency on constrained problems where the optimal front lies close to a narrow feasible boundary.

**Fig 1:**
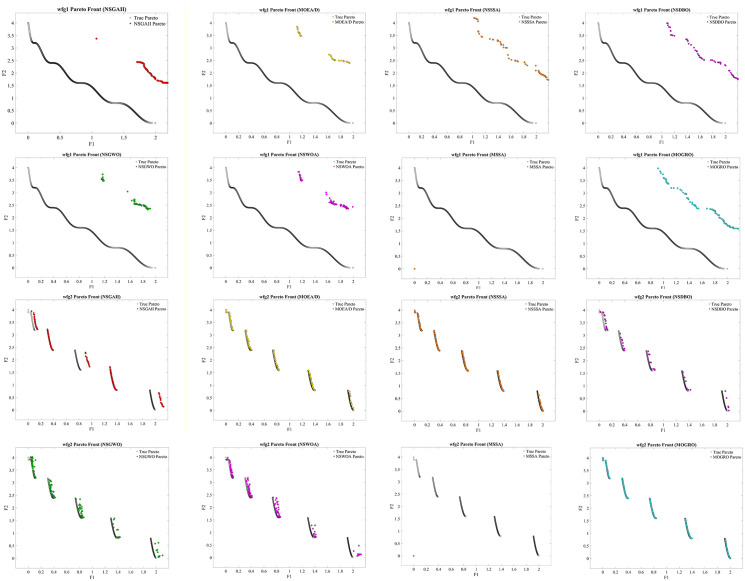
Comparison of the Pareto front distribution (1).

**Fig 2:**
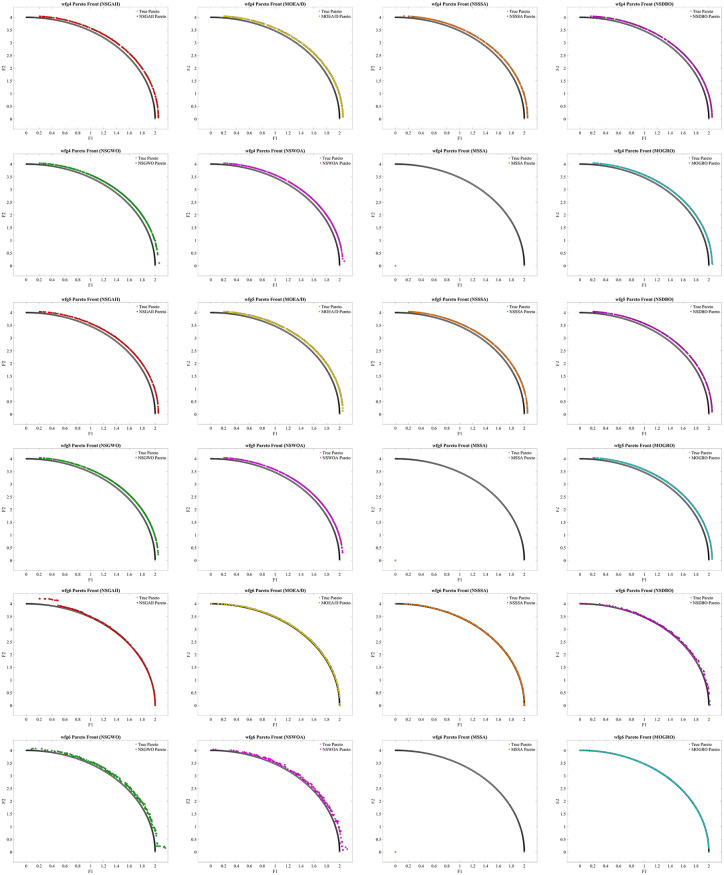
Comparison of the pareto front distribution (2).

**Fig 3:**
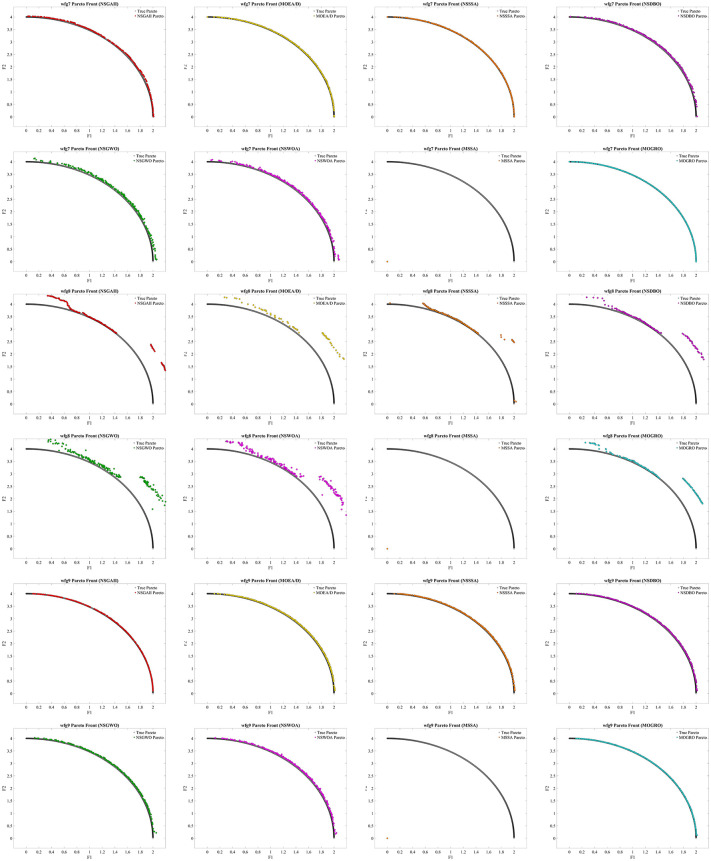
Comparison of the Pareto front distribution (3).

**Fig 4:**
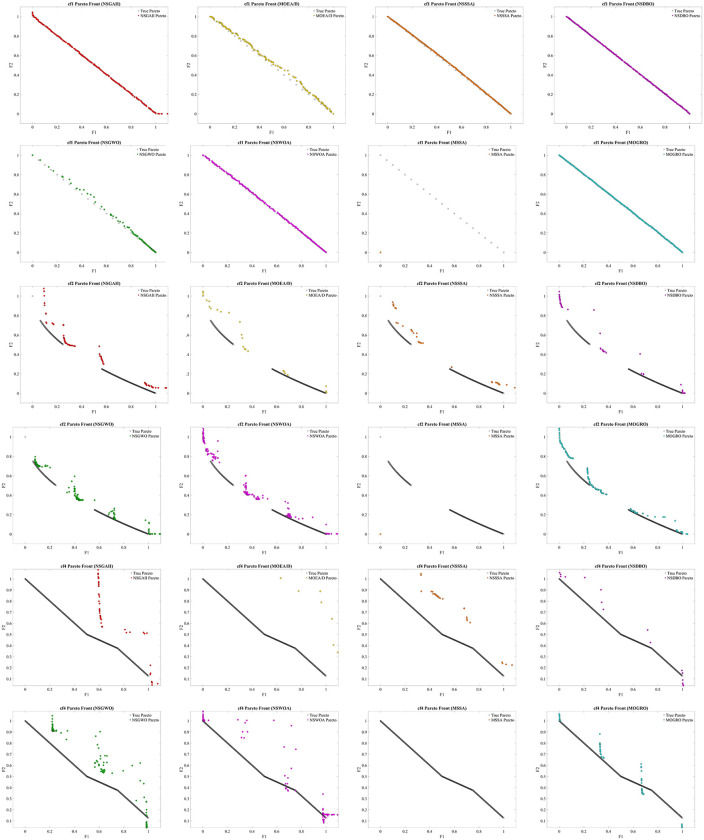
Comparison of the Pareto front distribution (4).

**Fig 5:**
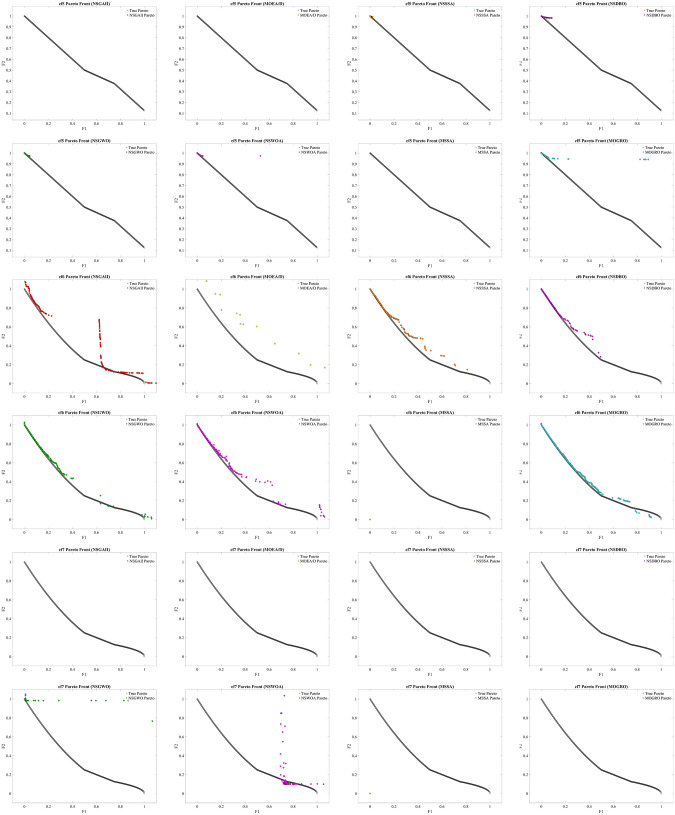
Comparison of the Pareto front distribution (5).

**Fig 6:**
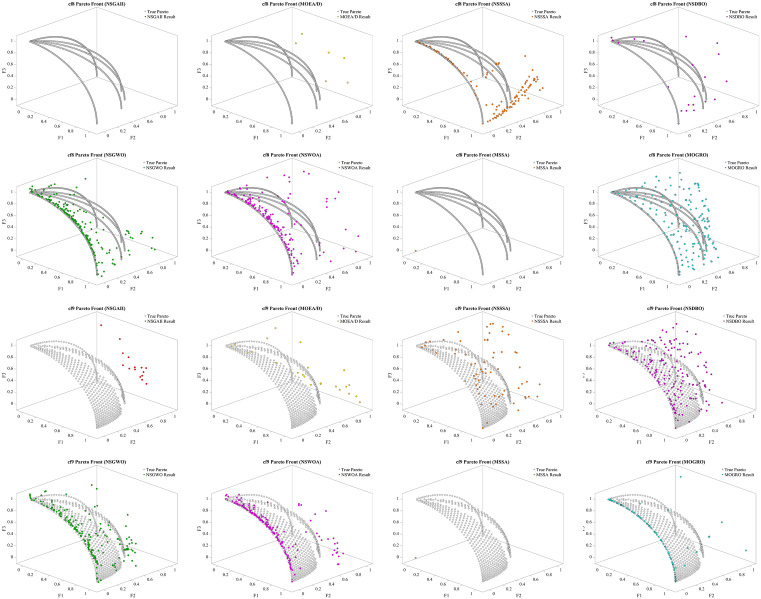
Comparison of the Pareto front distribution (6).

**Fig 7:**
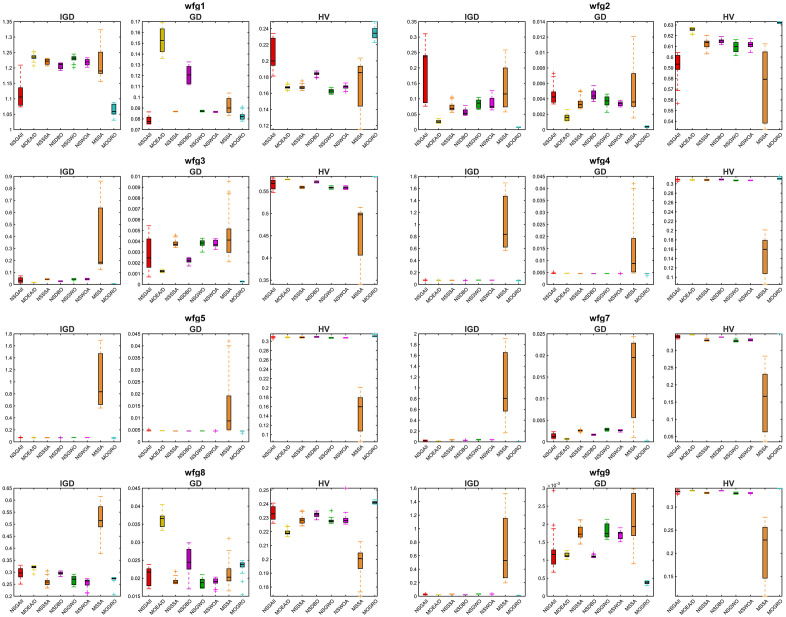
The box plots produced by algorithms for stated problems (1).

**Fig 8:**
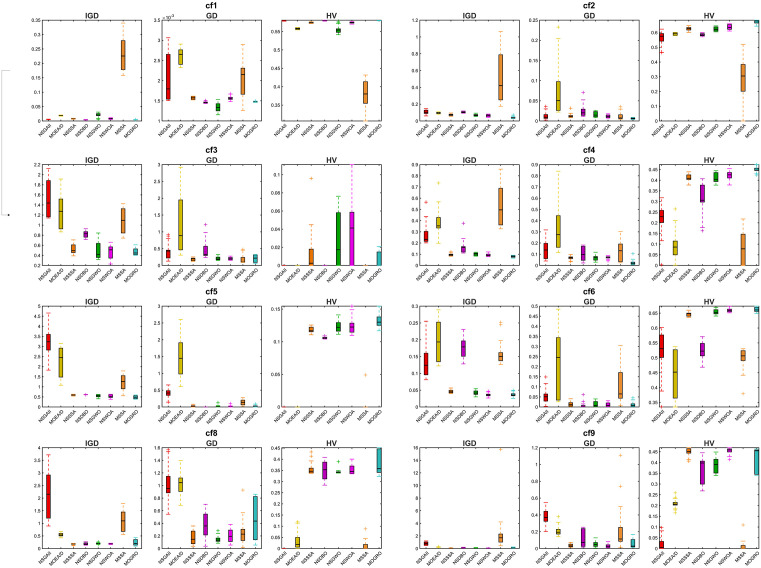
The box plots produced by algorithms for stated problems (2).

To evaluate MOGRO’s convergence and diversity, Figs 1–6 illustrate the optimal Pareto fronts generated by MOGRO and the seven comparison algorithms alongside the true Pareto fronts of each test function. It can be clearly observed that the Pareto fronts produced by MOGRO are closer to the true Pareto optimal solutions. Figs 7 and 8 present box plots of the IGD, GD, and HV metrics over 30 independent runs for each problem. These box plots demonstrate that MOGRO consistently achieves better and more stable performance across most test cases compared to the other algorithms.

In summary, based on the rankings of IGD, GD, and HV, the p-value results from the Wilcoxon test, the Pareto front distribution, and the box plot analysis, it can be concluded that the proposed MOGRO exhibits a significant advantage over the other seven algorithms.

Overall, the experimental results on the benchmark test suite clearly demonstrate the superiority and robustness of the proposed MOGRO algorithm. From the perspective of convergence, MOGRO consistently achieves lower IGD and GD values across most test problems, indicating that the obtained solution sets are closer to the true Pareto front. In terms of diversity, the HV results show that MOGRO is capable of generating well-distributed Pareto fronts, effectively covering the objective space. This performance can be attributed to the synergistic effect of the reference-point-guided environmental selection and the external archive mechanism, which jointly enhance both convergence accuracy and solution diversity.

Furthermore, MOGRO maintains stable performance across benchmark problems with different characteristics, including separable and non-separable functions, multimodal landscapes, and constrained optimization scenarios. This indicates that the proposed algorithm possesses strong adaptability and generalization capability when handling complex multi-objective optimization problems. Compared with the other seven state-of-the-art algorithms, MOGRO achieves superior average rankings across all evaluation metrics, which further confirms its effectiveness as a competitive multi-objective optimization framework.

### 3.5 Discussion of performance behavior and limitations

Although MOGRO achieves the best overall ranking across IGD, GD, and HV, the results should be interpreted from both successful and less successful cases. The strong performance of MOGRO on most WFG and CF problems can be attributed to the cooperation between its search operators and multi-objective selection mechanisms. The migration operator guides the population toward promising regions, the mining operator strengthens local exploitation, and the cooperation operator promotes information exchange among individuals. Meanwhile, the reference-point-guided environmental selection improves the distribution of solutions in the objective space, and the external archive prevents high-quality non-dominated solutions from being lost during population evolution. These mechanisms jointly explain why MOGRO performs well on multimodal, deceptive, non-separable, and constrained problems.

Nevertheless, the results also reveal several limitations. On WFG8, MOGRO does not obtain the best IGD, GD, or HV values. This problem involves strong dependency among decision variables, which may weaken the effectiveness of the standard migration and cooperation operators because variable interactions make independent position updates less efficient. MOEA/D performs better on this problem, indicating that decomposition-based local search may be more suitable for highly coupled variable structures. Similarly, on CF5, MOGRO does not achieve the best HV or IGD ranking. Since CF5 has multiple constraints and a relatively regular linear Pareto front, algorithms with stronger boundary exploitation may converge more directly to the feasible optimal region. In contrast, MOGRO tends to preserve a wider set of candidate solutions through reference-point guidance and archive maintenance, which improves diversity but may slightly reduce convergence efficiency when the feasible Pareto region is narrow and regular.

These observations indicate that MOGRO is particularly suitable for problems requiring a balance between convergence and distribution, especially those with complex Pareto front shapes, multimodality, deceptive landscapes, and constrained feasible regions. However, for problems with strong variable dependency or narrow linear feasible boundaries, additional mechanisms such as adaptive decomposition, constraint-boundary learning, or variable-correlation modeling may further improve its performance. Therefore, the results not only demonstrate the superiority of MOGRO but also clarify the conditions under which its advantages are most evident and where future improvements are needed.

## 4 UAV path planning performance test comparison

UAV path planning was selected as the real-world application scenario because it naturally involves conflicting objectives and practical safety constraints. In actual UAV missions, such as disaster rescue, regional inspection, and communication maintenance, a shorter path can reduce flight time and energy consumption, but it may increase the probability of passing close to obstacles or threat regions [69,70]. Conversely, a safer path may increase the total flight distance. Therefore, UAV path planning is not a simple single-objective shortest-path problem, but a typical multi-objective optimization problem that requires a set of trade-off solutions for decision-makers [71].

In this study, the UAV path planning model considers two conflicting objectives: path length and obstacle threat cost. Although the current simulation does not fully include dynamic obstacles, wind disturbance, communication uncertainty, or sensor noise, it provides a controlled and reproducible validation environment for examining whether MOGRO can generate diverse and high-quality path alternatives under different obstacle distributions. The symmetric and asymmetric obstacle scenarios are designed to represent different spatial risk distributions. Symmetric scenarios mainly test whether the algorithm can maintain stable solution distribution when obstacles are regularly arranged, whereas asymmetric scenarios better reflect irregular real-world environments where threats are unevenly distributed.

The experimental results show that MOGRO can generate a more uniformly distributed Pareto front in most UAV path planning scenarios, providing multiple feasible path choices with different trade-offs between flight distance and safety. This is important in practical UAV missions because decision-makers may select different solutions according to mission priorities. For example, in emergency rescue, a shorter path may be preferred when time is critical; in hazardous inspection, a safer path with lower threat cost may be more appropriate. Therefore, the UAV experiment demonstrates the practical value of MOGRO in supporting flexible decision-making for complex engineering optimization problems.

### 4.1 Mathematical modeling of UAV path planning problem

#### 4.1.1 Path cost.

The primary objective of three-dimensional path planning for unmanned aerial vehicles (UAVs) is to find the shortest flight path between the take-off point and the target point. Generally, the UAV’s flight path points are denoted as $W_{ij}=\left(x_{ij},y_{ij},z_{ij}\right)$, representing the UAV’s position in three-dimensional space at the jth point of the ith flight path. The entire flight path $X_{i}$ can thus be expressed as a three-dimensional array containing n path points. The Euclidean distance between two consecutive path points is denoted as the path segment $\left\|\vec{W_{ij}W_{i,j+\text{1}}}\right\|$. Accordingly, the UAV flight path cost function $F_{\text{1}}$ is defined as shown in Eq (17):


$$\begin{array}{c} \begin{array}{c} F_{\text{1}}\left(X_{i}\right)=\sum_{j=\text{1}}^{n-\text{1}}\left\|\vec{W_{ij}W_{i,j+\text{1}}}\right\| \end{array} \end{array}$$ (17)

#### 4.1.2 Obstacle threat cost.

**Fig 9:**
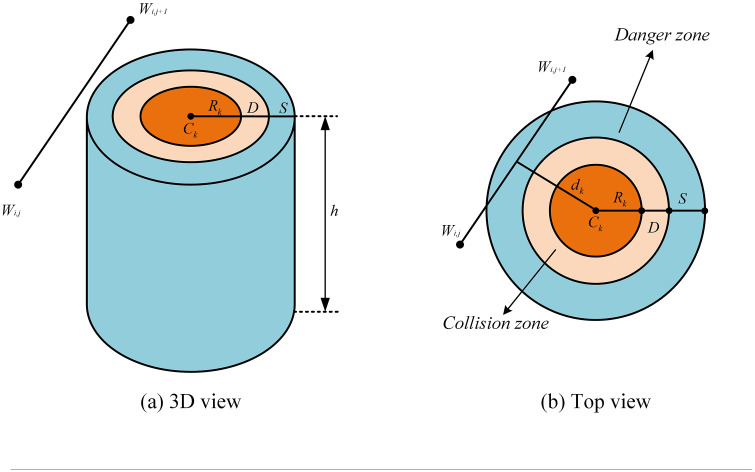
Illustration of UAV obstacle threat cost.

In UAV three-dimensional path planning, obstacle avoidance is a core requirement to ensure flight safety. In this study, cylindrical models are used to represent obstacle threat zones, with their 3D spatial form shown in Fig 9(a) and the corresponding 2D projection shown in Fig 9(b). Specifically, let the center of the kth cylindrical obstacle be denoted as $C_{k}$, and its radius as $R_{k}$. The design of the UAV obstacle avoidance threat cost function follows this principle: as the Euclidean distance $d_{k}$ between the UAV flight path segment $\left\|\vec{{\text{W}}_{\text{ij}}{\text{W}}_{\text{i},\text{j}+\text{1}}}\right\|$ and the obstacle center $C_{k}$ decreases, the threat cost increases inversely. This mechanism enforces the generation of safe trajectories that steer the UAV away from obstacles during the path optimization process.

In the UAV path planning problem, let the set of obstacle threat zones in the flight environment be $T=\{T_{\text{1}},T_{\text{2}},...,T_{k}\}$, where each threat zone $T_{k}$ is represented by a cylindrical model (as previously defined). Based on this, a cost function F2 related to obstacle avoidance threats is constructed, with its mathematical expressions given in Eqs. (18) and (19):


$$\begin{array}{c} \begin{array}{c} F_{\text{2}}\left(X_{i}\right)=\sum_{j=\text{1}}^{n-\text{1}}\sum_{k=\text{1}}^{K}T_{k}\left(\vec{W_{ij}W_{i,j+\text{1}}}\right) \end{array} \end{array}$$ (18)

$$\begin{array}{c} \begin{array}{c} T_{k}\left(\vec{W_{ij}W_{i,j+\text{1}}}\right)=\left\{ \begin{array}{c} @l\text{0}\;d_{k}>R_{k}\\ \;\left(R_{k}/d_{k}\right)\;\text{0}<d_{k}<R_{k}\\ \infty \;d_{k}=\text{0} \end{array}\right. \end{array} \end{array}$$ (19)

where, $X_{i}$ denotes the ith candidate path, which is a three-dimensional sequence composed of n waypoints; $W_{ij}$ represents the ordered waypoints; K is the total number of cylindrical obstacles in the environment; $T_{k}$ is the threat cost function of the kth obstacle, used to evaluate the interaction risk between the path and the obstacle; $\vec{W_{ij}W_{i,j+\text{1}}}$ denotes a segment between two adjacent waypoints; $d_{k}$ quantifies the minimum distance from the path segment to the central axis of the kthobstacle, reflecting the spatial proximity between the path and the obstacle; $R_{k}$ is the effective threat radius of the obstacle, which includes both its physical radius and a safety margin, defining the boundary of the obstacle’s danger zone.

#### 4.1.3 Flight altitude threat cost.

**Fig 10:**
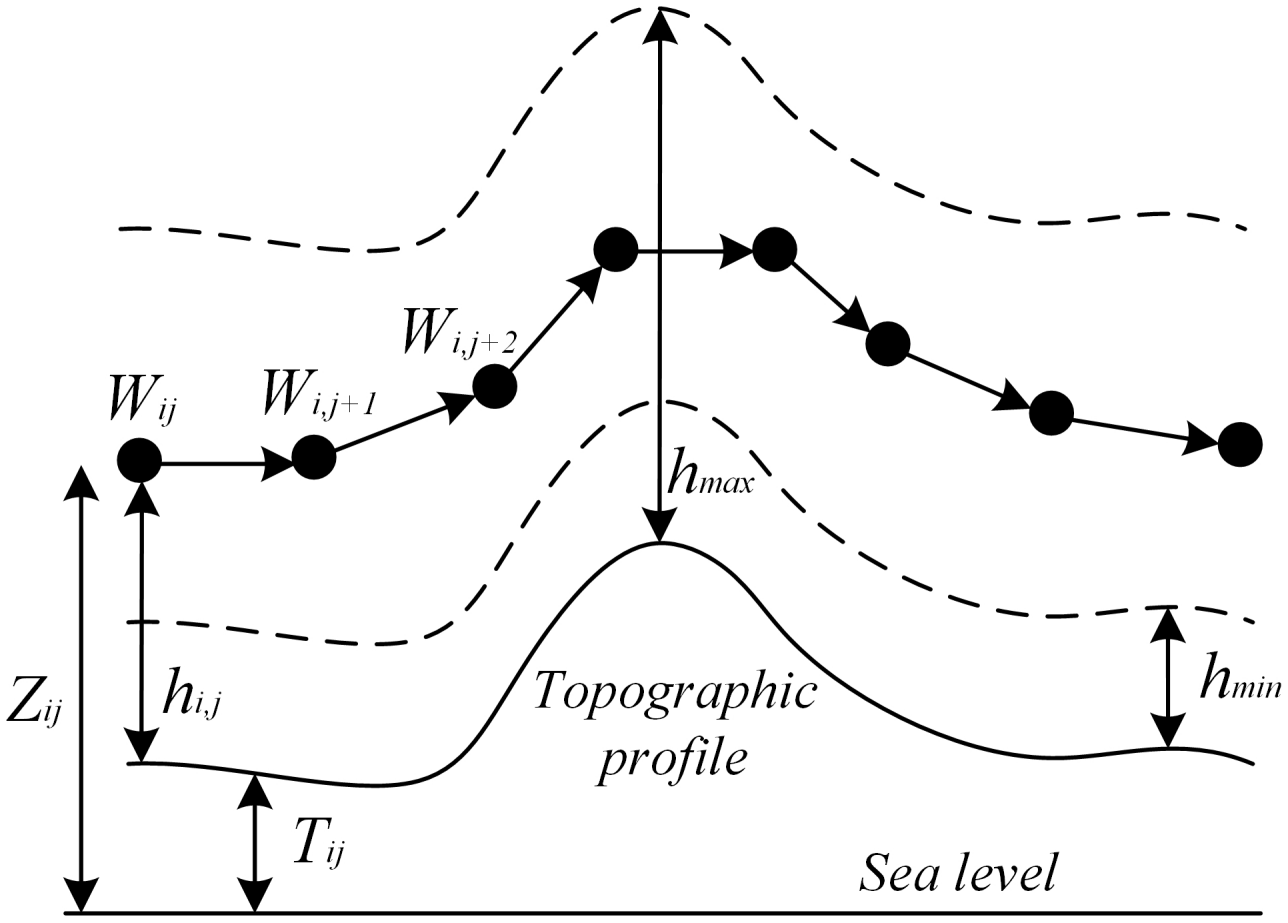
Illustration of UAV flight altitude threat cost.

In the UAV three-dimensional path planning process, strict altitude constraints must be observed, with the flight altitude restricted between a minimum height $h_{min}$ and a maximum height $h_{max}$, as shown in Fig 10. Here, $T_{ij}$ represents the terrain elevation at point (i,j) within the planning area, and $Z_{ij}$ denotes the UAV’s actual flight altitude relative to sea level at that position.

Let the UAV’s height above the reference terrain at path point $W_{ij}$ be denoted as $h_{ij}$, which is the difference between $Z_{ij}$ and $T_{ij}$. The cost function $H_{ij}$ related to the UAV at the current path point $W_{ij}$ is defined as shown in Eq. (20).


$$\begin{array}{c} \begin{array}{c} @cH_{ij}=\left\{ \begin{array}{c} @c\gamma_{h}\left(h_{ij}-h_{max}\right)\;\left(h_{ij}>h_{max}\right)\\ \text{0}\;\left(h_{min}<h_{ij}{<h}_{max}\right)\\ \gamma_{h}\left(h_{min}-h_{ij}\right)\;\left(\text{0}<h_{ij}{<h}_{min}\right)\\ \infty \;\left(h_{ij}<\text{0}\right) \end{array}\right. \end{array} \end{array}$$ (20)

Here, $H_{ij}$ represents the UAV flight altitude threat cost at the current path point $W_{ij}$. $\gamma_{h}$ is the penalty coefficient for the UAV’s flight altitude exceeding the constraint limits. $h_{ij}$ denotes the UAV’s flight altitude. $h_{min}$ and $h_{max}$ represent the minimum and maximum flight altitude constraints imposed on the UAV, respectively.

The cost function $F_{\text{3}}$ related to the UAV flight path can then be expressed by Eq. (21):


$$\begin{array}{c} \begin{array}{c} F_{\text{3}}\left(X_{i}\right)=\sum_{j=\text{1}}^{n}H_{ij} \end{array} \end{array}$$ (21)

#### 4.1.4 Flight corner threat costd.

**Fig 11:**
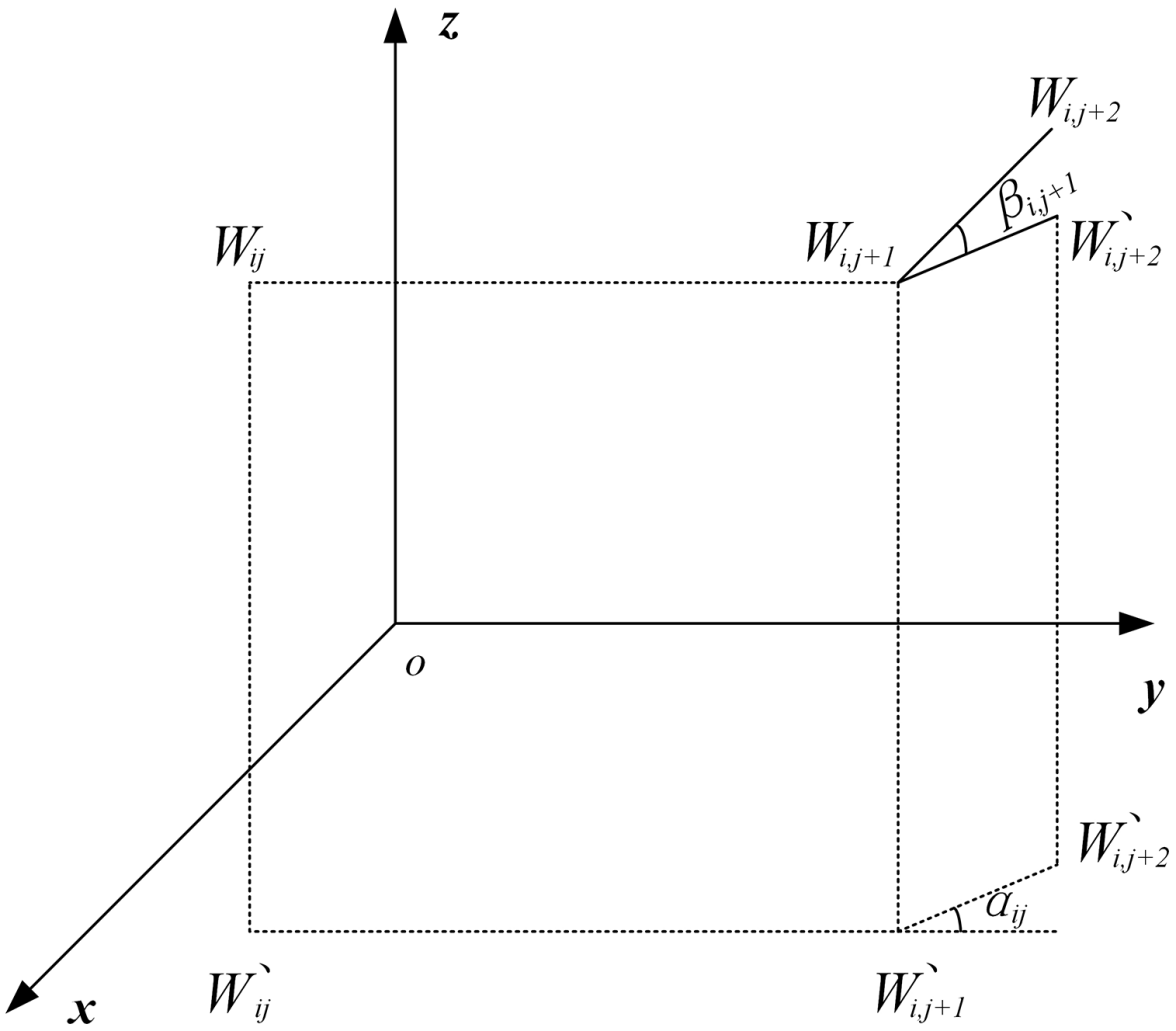
Illustration of UAV flight turn angle threat cost.

The UAV’s flight turning control parameters mainly include the horizontal turning angle and the vertical pitch angle. These two variables must comply with the UAV’s actual turning constraints; otherwise, the path planning model cannot generate a feasible flight trajectory. As shown in Fig 11, $\vec{W_{ij}W_{i,j+\text{1}}}$ and $\vec{W_{ij+\text{1}}W_{i,j+\text{2}}}$ represent two consecutive segments of the UAV’s flight path, while $\vec{\overset{\prime }{\underset{ij}{W}}\overset{\prime }{\underset{i,j+\text{1}}{W}}}$ and $\vec{\overset{\prime }{\underset{ij+\text{1}}{W}}\overset{\prime }{\underset{i,j+\text{2}}{W}}}$ are their projections onto the x–y plane.

Let k be the unit vector in the axial direction, then the calculation formula of the vector $\vec{\overset{\prime }{\underset{ij+\text{1}}{W}}\overset{\prime }{\underset{i,j+\text{2}}{W}}}$, as well as the formulas for the horizontal turn angle $\alpha_{ij}$ and vertical pitch angle $\beta_{i,j+\text{1}}$, are given by Eqs. (22)–(24):


$$\begin{array}{c} \begin{array}{c} \overset{\prime }{\underset{ij+\text{1}}{W}}\overset{\prime }{\underset{i,j+\text{2}}{W}}=k\times \left(\vec{W_{ij}W_{i,j+\text{1}}}\times k\right) \end{array} \end{array}$$ (22)

$$\begin{array}{c} \begin{array}{c} \alpha_{ij}=arctan\left(\frac{\vec{\overset{\prime }{\underset{ij}{W}}\overset{\prime }{\underset{i,j+\text{1}}{W}}}\times \vec{\overset{\prime }{\underset{ij+\text{1}}{W}}\overset{\prime }{\underset{i,j+\text{2}}{W}}}}{\vec{\overset{\prime }{\underset{ij}{W}}\overset{\prime }{\underset{i,j+\text{1}}{W}}}\cdot \vec{\overset{\prime }{\underset{ij+\text{1}}{W}}\overset{\prime }{\underset{i,j+\text{2}}{W}}}}\right) \end{array} \end{array}$$ (23)

$$\begin{array}{c} \begin{array}{c} \beta_{ij}=arctan\left(\frac{Z_{i,j+\text{1}}-Z_{ij}}{\left\|\vec{\overset{\prime }{\underset{ij}{W}}\overset{\prime }{\underset{i,j+\text{1}}{W}}}\right\|}\right) \end{array} \end{array}$$ (24)

where $\alpha_{ij}$ represents the UAV’s turning angle in the horizontal plane, and $\beta_{ij}$ represents the pitch angle in the vertical direction.

Meanwhile, let the penalty coefficients for violating the UAV’s horizontal turn angle and vertical pitch angle constraints be denoted as $a_{\text{1}}=\text{1}$ and $a_{\text{2}}=\text{1}$. Then, the cost function $F_{\text{4}}$ related to the UAV’s flight turn angles can be expressed as Eq. (25):


$$\begin{array}{c} \begin{array}{c} F_{\text{4}}\left(X_{i}\right)=a_{\text{1}}\sum_{j=\text{1}}^{n-\text{2}}\alpha_{ij}+a_{\text{2}}\sum_{j=\text{1}}^{n-\text{1}}\left|\beta_{ij}-\beta_{i,j+\text{1}}\right| \end{array} \end{array}$$ (25)

#### 4.1.5 Objective function for 3D path planning of UAV.

Considering the shortest path cost, minimum threat cost, flight altitude cost, and flight turn angle cost associated with the UAV flight path XX, a multi-objective function based on multi-factor constraints is constructed. The first objective function $f_{\text{1}}$ represents the shortest path cost, and the second objective function $f_{\text{2}}$ represents the minimum threat cost, which is the sum of obstacle threat cost, flight altitude threat cost, and flight turn angle threat cost. The detailed definitions of the two objective functions are given in Eqs. (26) and (27):


$$\begin{array}{c} \begin{array}{c} f_{\text{1}}\left(X_{i}\right)=F_{\text{1}}\left(X_{i}\right) \end{array} \end{array}$$ (26)

$$\begin{array}{c} \begin{array}{c} f_{\text{2}}\left(X_{i}\right)=F_{\text{2}}\left(X_{i}\right)+F_{\text{3}}\left(X_{i}\right)+F_{\text{4}}\left(X_{i}\right) \end{array} \end{array}$$ (27)

#### 4.1.6 UAV path planning experimental scenario design.

To thoroughly evaluate the performance of each algorithm in the multi-objective UAV path planning problem, this study, based on the previously established optimization model, systematically designed four types of typical obstacle avoidance test scenarios: symmetric and asymmetric obstacle avoidance scenarios with 8 obstacles and with 10 obstacles. All obstacles are designed as cylinders with a certain height. All scenarios are set within a 1000 m × 800 m flight area on the xoy plane. The 8-obstacle scenarios adopt a flight path configuration from the starting point [50,50,150] to the endpoint [800,580,100], while the 10-obstacle scenarios adopt a path from [160,60,150] to [880,720,100], with each scenario uniformly set with 15 intermediate optimization nodes. The geometric parameters of obstacles in the 8-obstacle symmetric and asymmetric avoidance scenarios are listed in Table 10, while those for the 10-obstacle symmetric and asymmetric avoidance scenarios are presented in Table 11. The tables provide detailed information on each obstacle’s position coordinates, radius, height, and other relevant parameters.

**Table 10:** Parameter settings for drone obstruction with 8 Obstacle.

Scene 1:8 Obstacle symmetrical scene				Scene 2: 8 Obstacle asymmetric scene			
Threat area	coordinate (𝐱,𝐲)	Height/m	Threat radius/m	Threat area	coordinate (𝐱,𝐲)	Height/m	Threat radius/m
1	200,200	120	40	1	180,220	120	55
2	200,400	120	50	2	220,380	120	65
3	200,600	120	60	3	250,550	120	75
4	425,300	120	70	4	380,280	120	45
5	425,500	120	80	5	420,480	120	85
6	650,200	120	40	6	580,180	120	60
7	650,400	120	50	7	620,420	120	70
8	650,600	120	60	8	650,580	120	50

**Table 11:** Parameter settings for drone obstruction with10 Obstacle.

Scene 3:10 Obstacle symmetrical scene				Scene 4: 10 Obstacle asymmetric scene			
Threat area	coordinate (x,y)	Height/m	Threat radius/m	Threat area	coordinate (x,y)	Height/m	Threat radius/m
1	300,300	150	80	1	400,500	100	80
2	740,300	150	70	2	600,200	150	70
3	400,500	150	70	3	500,350	150	70
4	640,500	150	50	4	350,200	150	50
5	250,650	150	50	5	700,550	150	50
6	790,650	150	80	6	650,750	150	80
7	520,400	150	70	7	750,350	150	70
8	520,600	150	70	8	250,350	150	70
9	350,200	150	70	9	530,620	150	70
10	690,200	150	60	10	850,200	150	60

### 4.2 Algorithm performance comparison experiment

**Fig 12:**
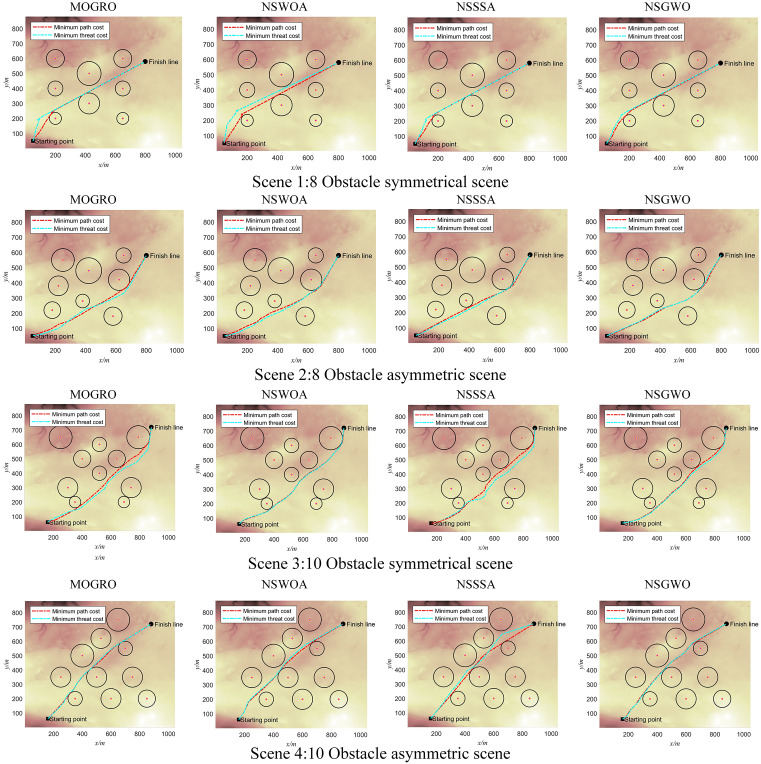
UAV route projection maps obtained under different obstacle avoidance scenarios.

**Fig 13:**
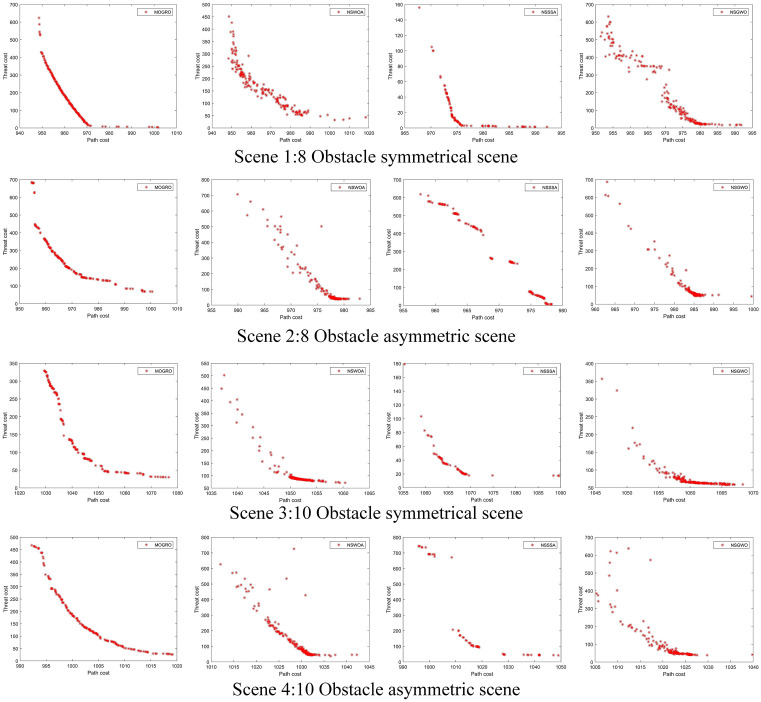
Pareto fronts obtained under different obstacle avoidance scenarios.

As shown in Fig 12 presents the UAV route projection maps of the minimum path cost and minimum threat cost obtained by the four best-performing algorithms—MOGRO, NSWOA, NSSSA, and NSGWO—in the UAV multi-objective path planning problem under different obstacle avoidance scenarios. Fig 13 shows the distribution of the Pareto fronts obtained by these four algorithms in the various obstacle avoidance scenarios.

Although the UAV route projection maps optimized by the four algorithms under different obstacle avoidance scenarios allow us to visually observe the UAV paths obtained, it is difficult to directly assess the performance superiority of each algorithm in the multi-objective UAV path planning problem solely from these projections. However, the distribution of the Pareto fronts obtained by the four algorithms across different obstacle avoidance scenarios reveals that, in almost all scenarios, the proposed MOGRO achieves a more uniform distribution of Pareto front solutions. The only exception is Scenario 1—the 8-obstacle symmetric avoidance scenario—where the solution distribution uniformity is slightly inferior to that of NSSSA. Therefore, based on the distribution of solutions, we can conclude that the proposed MOGRO outperforms other advanced algorithms by obtaining a wider range of high-quality solutions in the multi-objective UAV path planning problem. This indicates that MOGRO can provide more practical options for real-world UAV optimization issues. It demonstrates superior performance in terms of the distribution quality of solutions obtained for the multi-objective UAV optimization problem, significantly surpassing other algorithms.

To compare the quality of solutions obtained by all algorithms, Table 12 presents the optimal path costs and optimal threat costs achieved by each algorithm under different obstacle avoidance scenarios, along with their corresponding numerical rankings (a smaller value indicates a higher rank, and the top-ranked results are highlighted in bold). Here, m.pc denotes the minimum path cost obtained by the algorithm, and m.tc denotes the minimum threat cost obtained. The statistical results show that the proposed MOGRO algorithm achieved the best performance in the vast majority of test scenarios, ranking first in minimum path cost across all scenarios and first in minimum threat cost rankings except for Scenario 1. Notably, in Scenario 1 (the 8-obstacle symmetric avoidance scenario), although MOGRO ranked second in minimum threat cost behind NSSSA by a narrow margin, it still demonstrated a significant advantage. These experimental results fully demonstrate that the MOGRO algorithm exhibits excellent performance in solving the multi-objective optimization problem of UAV three-dimensional path planning, with its overall performance significantly outperforming other comparative algorithms.

**Table 12:** The rankings of Minimum path cost and Minimum threat cost obtained by different algorithm.

Algorithms		NSGAII	MOEA/D	NSSSA	NSDBO	NSGWO	NSWOA	MSSA	MOGRO
	**m.pc**	9.5241E + 02	9.5069E + 02	9.6134E + 02	9.5170E + 02	9.5023E + 02	9.5338E + 02	9.5373E + 02	**9.4795E + 02**
**scene 1**	**rank**	5	3	8	4	2	6	7	**1**
	**m.tc**	8.8303E + 00	1.5076E + 02	**9.4838E-02**	6.1196E + 00	9.3737E + 00	9.0465E + 00	8.0847E + 01	3.3266E + 00
	**rank**	4	8	**1**	3	6	5	7	2
	**m.pc**	1.0021E + 03	9.7822E + 02	9.5663E + 02	9.8847E + 02	9.6313E + 02	9.7387E + 02	9.6300E + 02	**9.5346E + 02**
**scene 2**	**rank**	8	6	2	7	4	5	3	**1**
	**m.tc**	3.0391E + 02	2.8952E + 02	5.4610E + 01	2.6723E + 02	5.4828E + 01	5.5229E + 01	1.3164E + 02	**3.7718E + 01**
	**rank**	8	7	2	6	3	4	5	**1**
	**m.pc**	1.1234E + 03	1.0650E + 03	1.0532E + 03	1.1081E + 03	1.0417E + 03	1.0363E + 03	1.0568E + 03	**1.0232E + 03**
**scene 3**	**rank**	8	6	4	7	3	2	5	**1**
	**m.tc**	1.9432E + 02	2.9176E + 02	7.1478E + 01	1.7056E + 02	5.4050E + 01	6.7800E + 01	1.7274E + 02	**4.3036E + 01**
	**rank**	7	8	4	5	2	3	6	**1**
	**m.pc**	1.0724E + 03	1.0081E + 03	9.9593E + 02	1.0807E + 03	1.0183E + 03	1.0039E + 03	1.0140E + 03	**9.9498E + 02**
**scene 4**	**rank**	7	4	2	8	6	3	5	**1**
	**m.tc**	4.4994E + 02	3.9909E + 02	4.1856E + 01	3.0033E + 02	8.7579E + 01	6.1392E + 01	6.6630E + 01	**3.5623E + 01**
	**rank**	8	7	2	6	5	3	4	**1**

## 5 Conclusions and future directions

This study proposes an innovative Multi-Objective Gold Rush Optimization algorithm (MOGRO), achieving for the first time a multi-objective extension of the GRO algorithm. As the first multi-objective optimization algorithm under the GRO framework, MOGRO realizes multi-objective optimization capabilities through the introduction of multiple novel mechanisms. The algorithm combines GRO with a reference-point-based and non-dominated sorting environmental selection mechanism to effectively evaluate solution set quality and filter high-quality populations for the next generation iteration. At the same time, an external archive mechanism is employed to store non-dominated optimal solutions discovered during the algorithm’s run, ensuring the final output solution set possesses high-quality characteristics.

To comprehensively assess the performance advantages of MOGRO, this study designs a systematic comparative experimental scheme. On 18 standard test problems and a multi-objective UAV three-dimensional path planning problem, MOGRO is compared against seven mainstream algorithms: NSGA-II, MOEA/D, NSSSA, NSDBO, NSGWO, NSWOA, and MSSA. Experimental results indicate that on the standard test set, statistical analyses based on IGD, GD, and HV performance metrics show MOGRO exhibits significant advantages on most test problems, especially excelling in convergence speed and solution set diversity. In UAV path planning applications, MOGRO not only achieves better minimum path cost and minimum threat cost but also generates a wider coverage Pareto front, providing richer optimization options for practical engineering applications.

This study systematically validates MOGRO’s optimization performance by constructing a comprehensive evaluation system combining quantitative and qualitative analyses. Quantitatively, statistical results based on the three core indicators IGD, GD, and HV demonstrate MOGRO’s overall performance significantly surpasses that of comparative algorithms; qualitatively, analysis of Pareto front shapes and coverage tests further confirm its outstanding capability in handling complex multi-objective optimization problems. Experimental results demonstrate that MOGRO achieves the best average ranking (IGD: 1.111, GD: 1.444, HV: 1.074) among all compared algorithms, indicating superior convergence and diversity performance. Meanwhile, in the typical engineering application of multi-objective UAV 3D path planning, MOGRO shows obvious competitive advantages by obtaining superior optimal solution sets. These experimental results fully demonstrate that MOGRO possesses excellent adaptability and robustness, effectively addressing multi-objective optimization problems with diverse characteristics and showing broad prospects for engineering applications.

Despite the promising performance of MOGRO, several limitations should be acknowledged. First, the incorporation of the external archive and reference-point-guided mechanisms introduces additional computational overhead, which may affect efficiency in large-scale problems. Second, the current implementation is primarily evaluated in static environments, particularly in the UAV path planning application, where obstacle configurations remain fixed and do not fully reflect real-world dynamics. Third, the performance of MOGRO may be influenced by parameter settings, such as population size and archive size, and a more comprehensive sensitivity analysis is still required to establish robust parameter selection guidelines.

To address these limitations, future research will focus on four key directions to further enhance the performance and application value of MOGRO. Specifically, hybrid optimization strategies will be developed to improve convergence speed, solution diversity, and stability in complex optimization problems. In addition, intelligent learning mechanisms, such as reinforcement learning, will be introduced to construct adaptive parameter tuning frameworks, enabling the algorithm to dynamically adjust its search behavior according to problem characteristics and thus improve adaptability in dynamic environments. Furthermore, the integration of MOGRO with advanced artificial intelligence techniques, including transfer learning and meta-learning, will be explored to establish more efficient intelligent optimization paradigms and enhance both solution accuracy and computational efficiency. Finally, future studies will emphasize the extension of MOGRO to more realistic and complex engineering applications. In particular, the current UAV path planning validation will be extended to dynamic and uncertain scenarios by incorporating moving obstacles, wind disturbances, sensor uncertainty, energy consumption constraints, and real-time replanning mechanisms. Moreover, broader applications in complex engineering domains, such as energy system optimization, aerodynamic design, intelligent aircraft systems, and industrial neural network optimization, will be investigated to further promote the transition from theoretical development to practical deployment.
